# The Unique Chemistry of Eastern Mediterranean Water Masses Selects for Distinct Microbial Communities by Depth

**DOI:** 10.1371/journal.pone.0120605

**Published:** 2015-03-25

**Authors:** Stephen M. Techtmann, Julian L. Fortney, Kati A. Ayers, Dominique C. Joyner, Thomas D. Linley, Susan M. Pfiffner, Terry C. Hazen

**Affiliations:** 1 Department of Civil and Environmental Engineering, University of Tennessee, Knoxville, Tennessee, United States of America; 2 Center for Environmental Biotechnology, University of Tennessee, Knoxville, Tennessee, United States of America; 3 Department of Earth and Planetary Sciences, University of Tennessee, Knoxville, Tennessee, United States of America; 4 Ocean Lab, University of Aberdeen, Newburgh, Aberdeenshire, United Kingdom; 5 Department of Microbiology, University of Tennessee, Knoxville, Tennessee, United States of America; 6 Biosciences Division, Oak Ridge National Laboratory, Oak Ridge, Tennessee, United States of America; Universidade Federal do Rio de Janeiro, BRAZIL

## Abstract

The waters of the Eastern Mediterranean are characterized by unique physical and chemical properties within separate water masses occupying different depths. Distinct water masses are present throughout the oceans, which drive thermohaline circulation. These water masses may contain specific microbial assemblages. The goal of this study was to examine the effect of physical and geological phenomena on the microbial community of the Eastern Mediterranean water column. Chemical measurements were combined with phospholipid fatty acid (PLFA) analysis and high-throughput 16S rRNA sequencing to characterize the microbial community in the water column at five sites. We demonstrate that the chemistry and microbial community of the water column were stratified into three distinct water masses. The salinity and nutrient concentrations vary between these water masses. Nutrient concentrations increased with depth, and salinity was highest in the intermediate water mass. Our PLFA analysis indicated different lipid classes were abundant in each water mass, suggesting that distinct groups of microbes inhabit these water masses. 16S rRNA gene sequencing confirmed the presence of distinct microbial communities in each water mass. Taxa involved in autotrophic nitrogen cycling were enriched in the intermediate water mass suggesting that microbes in this water mass may be important to the nitrogen cycle of the Eastern Mediterranean. The Eastern Mediterranean also contains numerous active hydrocarbon seeps. We sampled above the North Alex Mud Volcano, in order to test the effect of these geological features on the microbial community in the adjacent water column. The community in the waters overlaying the mud volcano was distinct from other communities collected at similar depths and was enriched in known hydrocarbon degrading taxa. Our results demonstrate that physical phenomena such stratification as well as geological phenomena such as mud volcanoes strongly affect microbial community structure in the Eastern Mediterranean water column.

## Introduction

Microbial diversity and activity are strongly dependent upon the environments in which microbes live [1, 2]. Localized chemistry can strongly impact microbial community structure and function [3, 4]. Environmental factors such as nutrients and temperature are important constraints on the microbes that live in a particular setting [5–7]. More recently, physical factors such as currents and oceanographic water masses have been shown to be important in dictating localized community structure [8–14]. The Mediterranean Sea has a unique chemistry with the waters being characterized by high salinity (38–39 psu relative to 35 psu in the open ocean), elevated bottom water temperatures (12–13°C relative to 4°C at similar depths and latitudes in the Atlantic) and ultraoligotrophic conditions (extreme phosphate limitation) [15].

The waters of the Eastern Mediterranean are stratified with distinct water masses present at different depths [16]. The upper water mass in the Eastern Mediterranean is the Atlantic Water (AW) occupying depths down to around 150 m. Salinity of the AW increases as it flows from the Strait of Gibraltar (∼36.5 psu) to the Levantine Basin in the Eastern Mediterranean (∼38.6) [16]. Below the AW, at depths between 150–400 m, is the Levantine Intermediate Water (LIW). The LIW is characterized by temperatures around 15°C with high salinity (39 psu). LIW forms in the Levantine Basin of the Eastern Mediterranean and flows at intermediate depths east to west. The Eastern Mediterranean Deep Water (EMDW) occupies depths below 400 m. The water temperature of the EMDW stabilizes at approximately 13.5°C. The unique chemistry and complex oceanographic regimes of the Eastern Mediterranean may select for distinct microbial communities throughout the water column, which are adapted for growth in nutrient limited environments.

The microbial communities of the Northwestern and Northeastern Mediterranean have been studied to investigate how microbes are affected by the ultraoligotrophic conditions of the Mediterranean [17, 18]. For the most part these studies have focused on particular groups of microbes and not entire communities. Two studies focusing on phototrophs, indicated that microbes adapted for growth under low nutrient conditions dominate the phototrophic community in areas under nutrient limitation [17, 18]. Metagenomic analysis of one site in the Eastern Mediterranean showed that an increased number of proteins involved in phosphate transport and processing are an important adaptation to growth under phosphate limiting conditions [19].

It has been hypothesized that the skewed N:P ratio of the Eastern Mediterranean may be due to high rates of nitrogen fixation [20, 21]. However, multiple studies have demonstrated that nitrogen fixation rates are very low in the photic zone of Eastern Mediterranean waters [22, 23]. More recent studies demonstrated that nitrogen fixation is much higher in the aphotic zone of the Eastern Mediterranean, with a larger percentage of nitrogen fixation in the Eastern Mediterranean occurring in the deep water [24].

Additional studies have investigated the changes that occur in the microbial community throughout the water column. In general bacterial numbers decrease from mid 10^5^ cells/ml in the surface waters to mid 10^4^ cells/ml in the bottom waters [25]. Yokokawa *et al*. (2010) demonstrated that there are differences in bacterial abundance and microbial community structure at different depths [25]. However, there were also localized distinctions between samples collected within the same water masses from different sampling stations. It was suggested that these site-specific differences could be attributed to local chemical differences, such as organic matter load [25]. Most of the investigations into microbial community of the Eastern Mediterranean water column have focused on the Northeastern Mediterranean or off the coast of Israel. There is almost no information regarding the microbial community of the water column in the Southeastern Mediterranean, especially locations adjacent to the Nile River Delta.

Despite limited knowledge of the microbial community in the water column adjacent to the Nile River, several studies have investigated the microbial communities in the numerous natural hydrocarbon seeps in the Nile Deep Sea fan [26–29]. The Nile Deep sea fan is a sedimentary turbiditic system extending from the Nile delta into the Eastern Mediterranean [30]. Recent work has indicated that mud volcanoes are found throughout the Nile Deep-Sea Fan, the Olympia field on the Mediterranean ridge, and the Anaximander Mountains in the Northeastern Mediterranean [31, 32]. These natural seeps are hot spots of deep-sea life, and impact the diversity of microbes present in the seep-associated sediments [33]. Active mud volcanoes can emit large volumes of methane and other hydrocarbons and in turn have the potential to impact the microbial community of the water column overlaying these seeps.

Several studies have investigated the microbial community in mud volcano sediments throughout the Eastern Mediterranean [26, 27, 29]. These sediment communities are known to be diverse and contain a variety of microbes involved in sulfur oxidation, methanotrophy, methylotrophy, degradation of higher hydrocarbons, and anaerobic methane oxidation (AOM) [27–29]. In this study, we collected samples from the water column above the North Alex Mud Volcano in order to determine the effect of geological phenomena such as hydrocarbon seeps on structuring the microbial community of the water column.

The Eastern Mediterranean is known for the stratification of the water column and the presence of a number of active mud volcanoes. Our study aims to characterize the effect of water stratification and mud volcanism on the microbial community in the water column of the Southeastern Mediterranean. We combine chemical measurement with phospholipid fatty acid (PLFA) analysis and massively-parallel 16S rRNA sequencing to characterize microbial abundance and diversity at five stations. PLFA analysis provides a robust measure of microbial biomass as well as insights into the physiological state of the active microbial community in a sample [34]. Lipid biomarkers can also be used as indicators for the presence of particular microbial groups [35]. 16S rRNA sequencing provides an in-depth look into which microbial taxa are present with much finer taxonomic precision than can be achieved with PLFA analysis alone. These complementary techniques were used in order to reduce the biases that any single technique would have and provide multiple lines of evidence for any conclusion [36].

## Materials and Methods

### Site Description and Permitting

Samples were collected between 11 and 15 October 2012 at five stations in the West Nile Delta region of the Nile Deep Sea Fan aboard the MV Fugro Navigator. This work was conducted in BP’s West Nile Delta Concession. No specific permits were required for collection of these samples. These field studies did not include the collection of any endangered or protected species.

### Sample Collection

**Fig 1:**
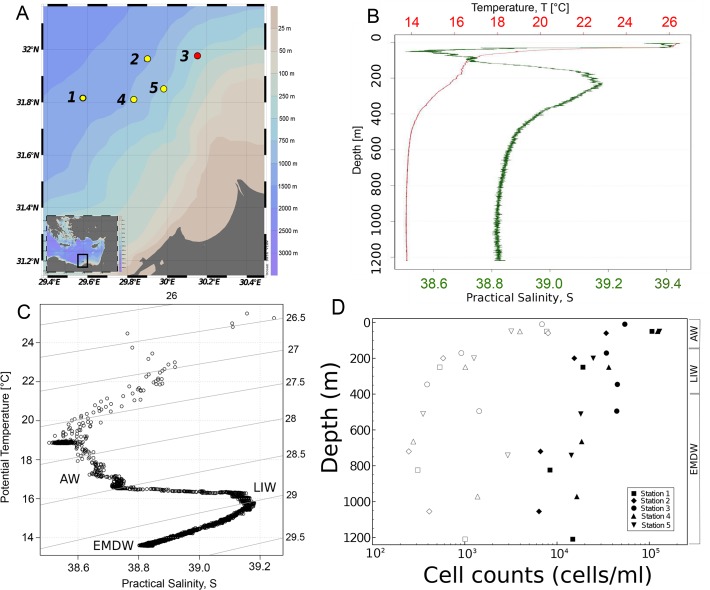
Site Characterization. (A) Map of sampling locations. Station 3 (red) is the North Alex Mud Volcano. Maps of sampling sites were prepared using the ODV software package [38]. (B) CTD profile of Temperature and salinity for site 1 (depth 1230 m). Temperature measured in °C is shown in red. Salinity measured in Practical Salinity Units (psu) is shown in green. (C) Annotated T-S plot with data from the CTD profile of site 1. Three distinct water masses are visible below the surface mixed layer. The AW is characterized by water with salinities between 38.6 and 38.8 psu and temperatures between 16–18°C. The LIW is characterized by high salinity around 39.2 psu and temperatures around 16°C. The EMDW is characterized by waters with salinities near 38.8 and temperatures near 14°C. Visualization of CTD data was performed in R[39] using the oce package [37]. (D) Cell counts as determined by AODC and PLFA as a function of depth. At each station samples were taken in each of the three water masses. Samples from each station are represented by different symbols. Cell counts as determined by AODC are indicated by closed symbols. Cell numbers as estimated by PLFA measurements are shown by open symbols. The depths corresponding to each water mass are marked on the right side of the plot. Biomass is reported at cells/ml of seawater.

Temperature, salinity, oxygen saturation, pH, and turbidity were measured at each station using a Valeport Midas+ CTD (Fig. 1B and S1 Fig.). Samples were taken from four depths based on the CTD profiles. Temperature and salinity profiles were constructed using the oce package in R [37]. One sample from each station was taken within or directly above the thermocline. One sample was taken within the region of increased salinity that occurred between 150 and 400 m. Another sample was taken at two-thirds of bottom depth. The fourth sample was collected 20 m above the sea floor. In total, 20 samples were collected (S1 Table). These stations represented diverse sea floor features, including the North Alex Mud Volcano and the Alexandria Canyon (Station 3 and 4 respectively, Fig. 1A).

Water was collected from each depth using Niskin bottles. 100 ml of water were frozen at −20°C for analysis of dissolved organic carbon (DOC) and inorganic nutrients. Forty ml of water were fixed in 4% formaldehyde and stored at 4°C for acridine orange direct counts (AODC). Samples for microbial community analysis were collected using the large volume Stand Alone Particle Sampler (SAPS, Challenger Oceanic, UK with controller, battery and pump upgrades by Oceanlab, University of Aberdeen, Scotland). Between 62 and 123 L of seawater were filtered at depth through a 292 mm diameter nylon filter with a pore size of 0.2 μm (volume filtered for each sample is listed in S1 Table). The filter was sectioned into thirds, one-third for DNA analysis, one-third for PLFA analysis, and one-third as an archive.

### Geochemical Measurements

DOC, total dissolved nitrogen (TDN), and inorganic nutrients were measured at the SOEST Laboratory for Analytical Biogeochemistry (University of Hawaii). DOC and TDN were measured using a Shimadzu High-Temperature TOC-L Combustion Analyzer (Shimadzu, Japan). DOC is reported as non-purgeable organic carbon (NPOC). Quality control testing for NPOC and TDN was conducted using purchased Deep Seawater Reference Material (DSRM) from the RSMAS Consensus Reference Materials (CRM) Project (http://yyy.rsmas.miami.edu/groups-/biogeochem/CRM.html). Ammonia was measured fluorometrically following the method of Kerouel and Aminot (1997) [40]. Nitrate and nitrite were analyzed via the diazo reaction based on the methods of Armstrong *et al* (1967) [41] and Grasshoff (1983) [42]. Silicate measurement is based on the reduction of silicomolybdate in acidic solution to molybdenum blue by ascorbic acid [42]. Orthophosphate concentrations were determined based on the colorimetric method of Murphy and Riley (1962) [43].

In order to identify patterns and similarities in the physical and chemical parameters of samples from the same water mass, Principle Component Analysis (PCA) of environmental data was performed in R using the prcomp command [39]. The data was centered so that the variables are shifted to be zero centered and scaled by dividing each number by the standard deviation. PERMANOVA analysis [44] was performed on a Euclidian distance of the normalized environmental data using the Adonis function in Vegan [45].

### Acridine Orange Direct Cell Counts

AODC were performed as described previously [46]. Water samples for direct cell counts were preserved with 4% formaldehyde and stored at 4°C until processed. Cell counts were done with Zeiss Axioskop epifluorescence microscope (Carl Zeiss, Inc., Germany).

### PLFA Extraction and Analysis

One-third of the SAPS pump filter was stored at −80°C for PLFA analysis. The filter was transferred to a muffled glass centrifuge tube using a solvent-rinsed forceps. The total lipids were extracted using a two-phase extraction method with final ratio of methanol:chloroform:water buffer being 1:1:0.9 (v/v/v) and subsequently fractionated on a silicic acid column with only the polar lipids then transesterified into phospholipid fatty acid (PLFA) methyl esters [47]. The PLFA methyl esters were separated, quantified, and identified by gas chromatography–mass spectrometry (GC/MS) [47]. Archaeal lipids were not analyzed.

Cell counts were estimated from PLFA data using the conversion factor of 5.9 x 10^4^ cells per pmole of PLFA [34]. PLFA cell counts were compared with AODC counts to determine how microbial abundance changes with depth. AODC cell counts are able to detect all types of microbial cells including Eukaryotes, Archaea and Bacteria. Alternatively, PLFA analysis will only detect Eukaryotes and Bacteria. PLFA has the advantage of providing information on viable cells only [34, 48], whereas AODC will measure both viable and dead cells. The combination of these two methods provides a robust estimate of total and active prokaryotes in an environment.

In addition to biomass estimates, PLFAs were grouped into lipid classes. The mole percent of each lipid class was determined for samples from each water mass. To test the hypothesis that lipid classes were differentially abundant in each water mass, one-way ANOVA was performed comparing the mole percent of each lipid class in samples grouped according to water mass. P values were corrected using the false discovery rate calculation in R. Tukey honest significant difference test was used to determine which water masses were significantly different from each other. The full list of lipids in each sample is presented in S2 Table.

### DNA Extraction, Sequencing, Analysis

One-third of the SAPS filter was stored at −80°C for DNA analysis. DNA was extracted using a modified Miller DNA extraction method [49]. Quality of extracted DNA was determined by measuring 260/280 and 260/230 ratios on a NanoDrop spectrophotometer (Thermo Scientific, Waltham, MA). Concentration of DNA was determined using picogreen (Life Technologies, Carlsbad CA). The V4 region of the 16S rRNA gene was amplified using Phusion DNA polymerase (Thermo Scientific, Waltham, MA) with universal primers 515f and barcoded 806r, which are able to amplify both Bacterial and Archaeal sequences. Sequencing was performed on the Illumina MiSeq according to the protocol in Caporaso *et al* (2012) [50]. The resulting DNA sequences were analyzed using the QIIME version 1.8.0-dev pipeline [51]. Paired-end raw reads were assembled using fastq-join [52]. The assembled sequences were demultiplexed and quality filtered in QIIME to remove reads with phred scores below 20 (-q 19). Chimera detection was then performed on assembled reads using UCHIME [53, 54]. Assembled, quality-filtered and chimera checked sequences were deposited at MG-RAST (http://metagenomics.anl.gov/) (accession number 4571952.3–4571971.3). Sequences were then clustered into operation taxonomic units (OTUs, 97% similarity) with UCLUST [53] using the open reference clustering protocol. The resulting representative sequences were aligned using PyNAST [55] and given a taxonomic assignment using RDP [56] retrained with the May 2013 Greengenes release. The resulting OTU table was filtered to keep OTUs that were present at greater than 0.005%, and then rarified to 13,753 sequences per sample (the minimum number of remaining sequences in the samples). The alpha diversity of samples was determined using the Shannon, Simpson, and phylogenetic diversity (whole tree diversity) metrics. To test the hypothesis that alpha diversity was significantly different between these three water masses ANOVA and Tukey honest significant difference test were performed. Bray-Curtis dissimilarity [57], weighted and unweighted unifrac distances [58] were calculated from the rarefied OTU table using the beta_diversity.py script in QIIME.

### Statistical Analysis of Sequencing Data

In order to test the hypothesis that microbial communities from the same water mass were significantly similar to each other and statistically different from other water masses; hierarchical clustering and non-metric multidimensional scaling (NMDS) were used. Hierarchical clustering analysis was performed using the hclust command in the ecodist [59] package in R with a Bray-Curtis dissimilarity matrix using the average linkage method. To further test this hypothesis weighted Unifrac distances were used to construct two-dimensional NMDS plots. The lowest stress configuration was chosen from 50 iterations of plot construction. Stress values were calculated using the default stress calculation in the nmds command in the ecodist package. To test if samples from the same water mass were significantly different from each other PERMANOVA analysis was performed on both the Bray Curtis and weighted Unifrac matrices using the Adonis function in the vegan package in R. Samples were grouped according to the water mass from which the samples were taken using the depth cutoffs as follows: AW: 10–150 m, LIW: 150–300 m, EMDW: 300–1210 m. There were five samples in the AW group, five samples in the LIW group and ten samples in the EMDW group. PERMANOVA analysis was performed using 999 permutations. PERMANOVA analysis of all three groups will indicate if there is a significant difference between all three groups. To distinguish which water masses are different from each other, PERMANOVA analysis was done on subsets of the weighted Unifrac distance matrix that only include samples from two of the three water masses. These pair-wise PERMANOVAs were used to distinguish which water masses were different from each other.

To test if bacteria and archaeal populations were both stratified by water mass, the OTU table was split to separate OTUs identified as bacteria into one OTU table and OTUs identified as Archaea into another. Weighted Unifrac distances were determined for each of these domain-specific OTU tables. NMDS analysis was performed using weighted Unifrac distances as described above. To determine if the Bacterial and Archaeal communities in the three water masses were different from each other, PERMANOVA analysis was performed using the Adonis function in the vegan package in R.

Environmental variables were fit to the weighted Unifrac distance matrix in order to test which environmental variables explain the differences observed in the beta diversity analysis. Temperature, dissolved oxygen, depth, salinity, sulfate, silicate, nitrate, inorganic phosphate, NPOC, and total nitrogen were fit to the weighted Unifrac distance matrix using the envfit function in the vegan package in R. The significance of the variable fitting was determined using 999 permutations. Variables that fit the data with a p value of less than 0.05 were plotted.

In order to test the hypothesis that microbial classes are differentially abundant between these water masses, one-way ANOVA was performed to compare the relative abundance each taxonomic order in samples from different water masses. P values were corrected using False Discovery Rate correction. Tukey honest significant difference test was used as a post hoc test to identify in which water masses the taxa were differentially abundant. Taxonomic orders that had an ANOVA corrected p value of less than 0.05 were considered to be significantly different. To further identify microbial taxa that were indicative a particular water mass, Indicator species analysis was performed in R using the IndVal function in the labdsv package [60]. Indicator species analysis seeks to identify taxa that are present in the majority samples of one group and absent in the majority of samples from other groups. Indicator species analysis calculates an IndVal as described in Dufrene and Legendre [61]. IndVal is the product of the relative frequency and relative average abundance of a species or OTU in a cluster. The maximum IndVal of 100% is observed when an OTU is present in all sites of only one sample group. To test the significance of the IndVal, p values were calculated with 100 iterations, where in each iteration, the sample groupings were randomly assigned and an IndVal determined. These randomized IndVals were compared to the IndVal arrived at using the defined groupings to determine the likelihood of that IndVal being arrived at randomly. The p values for the IndVal calculation were corrected for multiple comparisons using the false discovery rate correction.

## Results

### Site Description

The temperature profiles of the water column indicated a thermocline at approximately 50 m depth (Fig. 1B). Below 400 m the water temperature became constant at 13.8°C. The salinity at these stations was between 38–39 psu. The dissolved oxygen remained high throughout the water column and decreased to about 70% of saturation at depth (Table 1). Turbidity and pH were relatively constant throughout the water column with pH at 8.2 and turbidity around 1.5 FTU. Temperature and salinity plots of the water profiles indicated the presence of three water masses at these sampling sites (Fig. 1 C).

**Table 1:** Physical and geochemical parameters. Samples collected within the three water masses were reported together. Numbers in bold represent the mean value for that parameter. Numbers in parentheses represent the range of values.

Parameter	AW (n = 5)	LIW (n = 5)	EMDW (n = 10)
**Temperature (°C)**	**20.2** (17.5–26.3)	**15.8** (15.5–16.0)	**13.9** (13.7–14.5)
**Salinity (psu)**	**38.8** (38.6–39.5)	**39.2** (39.1–39.2)	**38.8** (38.8–38.9)
**DO (% Saturation)**	**114.9** (102.9–127.5)	**89.8** (87.8–91.61)	**70.0** (65.4–76.4)
**pH**	**8.21** (8.20–8.22)	**8.20** (8.19–8.21)	**8.19** (8.18–8.20)
**Turbidity (FTU)**	**1.26** (1.19–1.31)	**1.25** (1.19–1.31)	**1.28** (1.19–1.38)
**Inorganic P (μmol/L)**	**0.0758** (0.008–0.171)	**0.139** (0.057–0.24)	**0.354** (0.298–0.437)
**Silicate (μmol/L)**	**0.4616** (0.377–0.576)	**1.98** (1.36–2.74)	**9.13** (6.65–9.98)
**Ammonia (μmol/L)**	**<0.01** (<0.01)	**<0.01** (<0.01)	**0.089** (0.028–0.131)
**Nitrate (μmol/L)**	**0.01** (0.01)	**2.67** (1.92–3.40)	**6.43** (5.86–6.95)
**Sulfate (mmol/L)**	**57.2** (35.3–74.9)	**49.9** (32.2–73.9)	**45.8** (22.9–74.9)
**Total N (μmol/L)**	**7.31** (7.08–7.72)	**8.48** (7.08–10.2)	**10.9** (9.42–13.9)
**NPOC (μmol/L)**	**97.4** (90.8–103.6)	**77.9** (61.6–88.6)	**71.0** (56.6–123.4)

### Physical and chemical parameters were similar for samples from the same water mass

**Fig 2:**
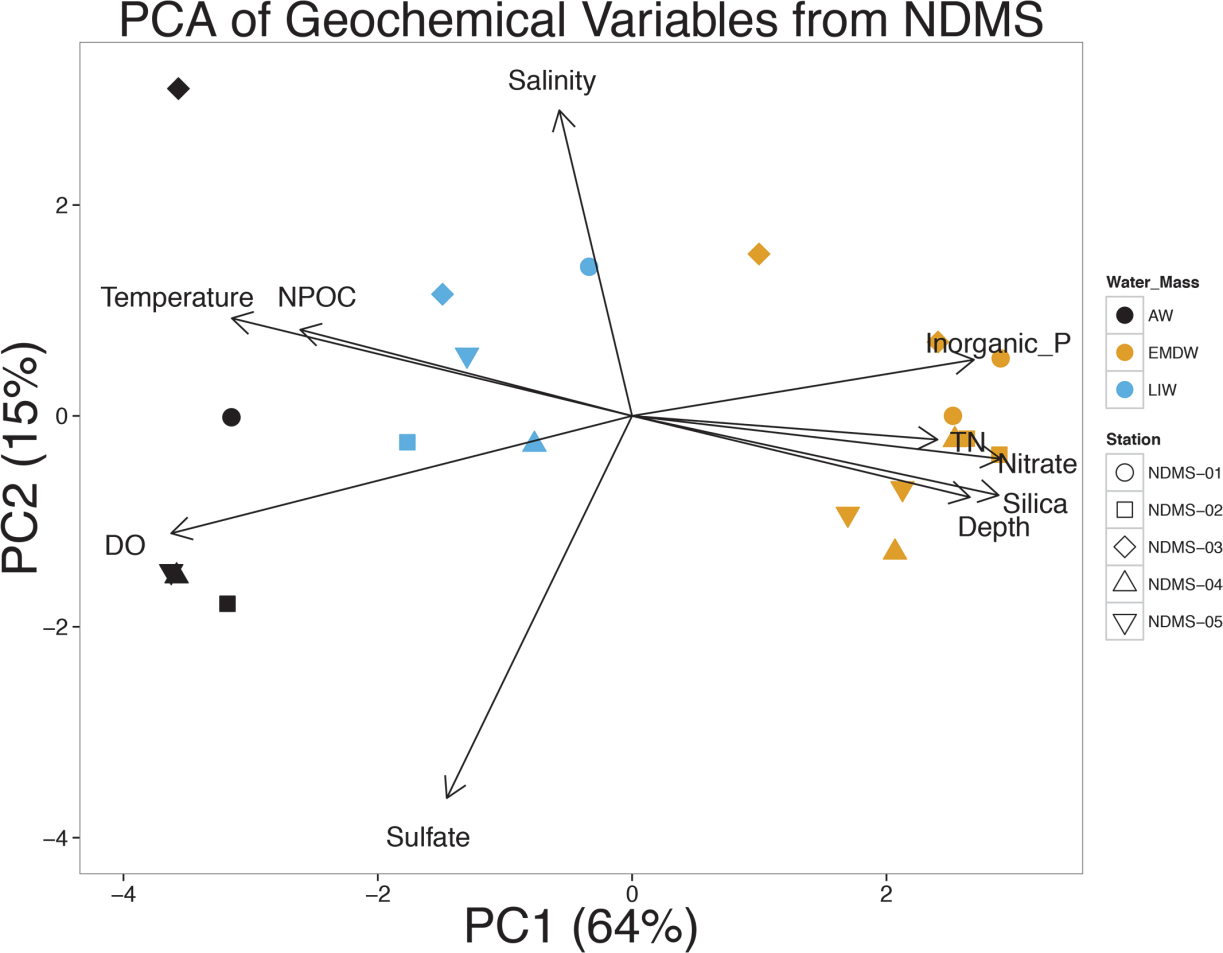
PCA analysis of physical and geochemical data. Salinity, inorganic phosphate, nitrate, ammonia, total nitrogen, silicate, temperature, salinity, sulfate, dissolved oxygen, and NPOC were used to construct the PCA plot. Color of symbols indicates the water mass from which that sample was taken (black corresponds to AW, blue corresponds to LIW, and orange corresponds to EMDW). Shape of symbols corresponds to the station from which they were taken. The percent explained by each of the depicted principle components are listed on the axis.

Water profiles of our sampling stations indicate that the salinity of the LIW is much higher than the AW and EMDW. Our data also show that the AW is characterized by low or below detection levels of inorganic nutrients, and high NPOC levels (mean 97.4 **μM**) (Table 1 and S1 Table). Inorganic nutrients concentrations increased in our samples throughout the LIW, whereas NPOC decreased with increasing depth (S2 Fig). Nutrient concentrations, NPOC, and salinity all remained fairly constant throughout the EMDW at these five sampling locations. PCA of environmental factors for the 20 water samples demonstrated that water samples from the same water mass cluster together on the PCA plot (Fig. 2). These distinct groupings corresponded to the three water masses. PERMANOVA of Euclidian distances demonstrated that samples from the same water mass were significantly different from samples from the other water masses (P_permutated_ = 0.001).

### Microbial abundance decreased with depth

Both AODC and PLFA biomass estimates indicate that the biomass was highest in the AW and gradually decreased with depth (Fig. 1D). Cell counts estimated by PLFA analysis were almost an order of magnitude lower than the cell numbers determined by AODC. Despite these discrepancies, both methods demonstrate that the microbial abundance in the AW was higher than in the intermediate and deep-water masses.

### Lipid classes were differentially abundant in each water mass

The mole percent of polyunsaturated lipids in our samples was significantly different between these three water masses (ANOVA P = 0.0004). Polyunsaturated lipids comprised 11.9 mole percent of the lipids in the AW, whereas they made up 2.7% and 1.9 mol% in the LIW and EMDW respectively (Table 2). Mid-Branched saturates were also differentially abundant between these water masses (ANOVA P = 0.02). The abundance of mid branched saturates was highest in the LIW and is significantly different from the abundance in the AW (Tukey HSD P = 0.005).

**Table 2:** PLFA data. Mean values of various PLFA categories are shown for the three water masses present in the Eastern Mediterranean.

PLFA groups (mol%)	AW (n = 5)	LIW (n = 5)	EMDW (n = 10)
Normal Saturates	53.4 (±7.6)	42.2 (±2.6)	34.4 (±16.8)
Terminally-Branched Saturates	1.17 (±0.72)	1.36 (±0.38)	1.15 (±1.0)
Mid-Branched Saturates	0.14 (±0.14)	4.17 (±2.4)	2.17 (±1.7)
Monosaturates	26.9 (±4.5)	29.7 (±6.2)	48.3 (24.3)
Cyclopropyl fatty acids	6.13 (±2.2)	19.7 (±1.2)	11.9 (±8.0)
Polyunsaturates	11.9 (±6.1)	2.72 (±3.8)	1.86 (±1.9)

### Microbial diversity is distinct for each water mass

**Fig 3:**
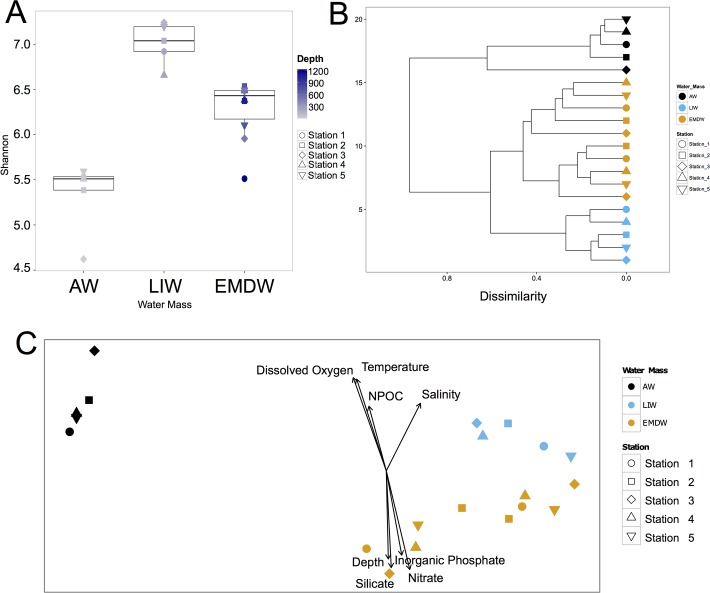
Microbial community analysis. (A) Box and whisker plot of Shannon diversity of the microbial community in each water mass. Shape of symbols corresponds to the station from which the samples were taken. Color of symbols corresponds to the depth from which the sample was taken. (B) Hierarchical clustering analysis of Bray-Curtis distances. Color of symbols corresponds to the water mass from which the samples were taken (black corresponds to AW, blue corresponds to LIW, and orange corresponds to EMDW). Shape of symbols corresponds to the station from which the samples were taken. (C) NMDS plot of weighted Unifrac distances. Color of symbols corresponds to the water mass from which the samples were taken (black corresponds to AW, blue corresponds to LIW, and orange corresponds to EMDW). Shape of symbols corresponds to the station from which the samples were taken. Vectors displaying the fit of environmental variables to the data are shown. Only vectors with p values of less than 0.05 are shown.

1.7 million 16S rRNA reads were retained after quality filtering. The average number of reads per samples was 83,948 with the number of reads ranging from 13,753 to 160,639 (S3 Table). The total number of OTUs present at a relative abundance of greater than 0.005% was 1,023. Diversity analysis using Shannon, Simpson, and Phylogenetic Diversity—whole tree metrics indicate that microbial diversity was lowest in the samples from the AW. The highest diversity was seen in samples from the LIW. Diversity then decreased in the EMDW (Fig. 3A and S3 Fig). Alpha diversity was significantly different between water masses for each of the metrics tested as determined by ANOVA and Tukey HSD test (Table 3).

**Table 3:** P values for ANOVA and Tukey HSD test comparing alpha diversity metrics for the three water masses in the Eastern Mediterranean.

	ANOVA		Tukey HSD Test		
Diversity Metric	ANOVA p value	ANOVA F Statistic	EMDW-AW	LIW-AW	LIW-EMDW
**Shannon**	1.53E-06	32.59	0.00018	9.88E-07	0.00248
**Simpson**	1.55E-06	32.50	0.00187	9.83E-07	0.00023
**PD Whole Tree**	3.93E-14	312.55	3.39E-13	3.74E-13	0.01529

Both hierarchical clustering and NMDS analysis indicated that samples from the same water mass clustered together (Fig. 3 B and C). There were significant differences in the microbial communities of each water masses as determined by PERMANOVA analysis (Bray Curtis—P_permuted_ = 0.001, weighted Unifrac—P_permuted_ = 0.001). Samples from AW clustered very closely together and were distant from samples derived from the deeper two water masses (Fig. 3B and 3C). PERMANOVA analysis indicates that the AW was significantly different from the LIW and EMDW (AW v. LIW—P_permutated_ = 0.006, AW v. EMDW—P_permutated_ = 0.001). PERMANOVA analysis comparing the LIW and EMDW showed that even though the differences between the LIW and EMDW were less pronounced, they were significant (P_permutated_ = 0.012). NMDS plots constructed using OTU tables separated by domain revealed that both the Bacterial and Archaeal communities clustered by water mass. PERMANOVA analysis of the separated microbial communities indicate that there were significant distinctions in the bacterial and archaeal communities between the three water masses (Archaea—P_permutated_ = 0.002; Bacteria—P_permutated_ = 0.001) (S4 Fig.).

Environmental variables were fit to the NMDS plot to examine which physical and geochemical factors affect community structure (Fig. 3C). Temperature, dissolved oxygen, salinity, silicate, nitrate, inorganic phosphate, NPOC, and total nitrogen all significantly fit the NMDS plot with p-values less than 0.05. The microbial community in the AW was strongly affected by NPOC and somewhat by temperature. Salinity partially dictates the distinction between the EMDW and LIW. Nutrient concentrations were the main factor structuring the community of the EMDW.

**Fig 4:**
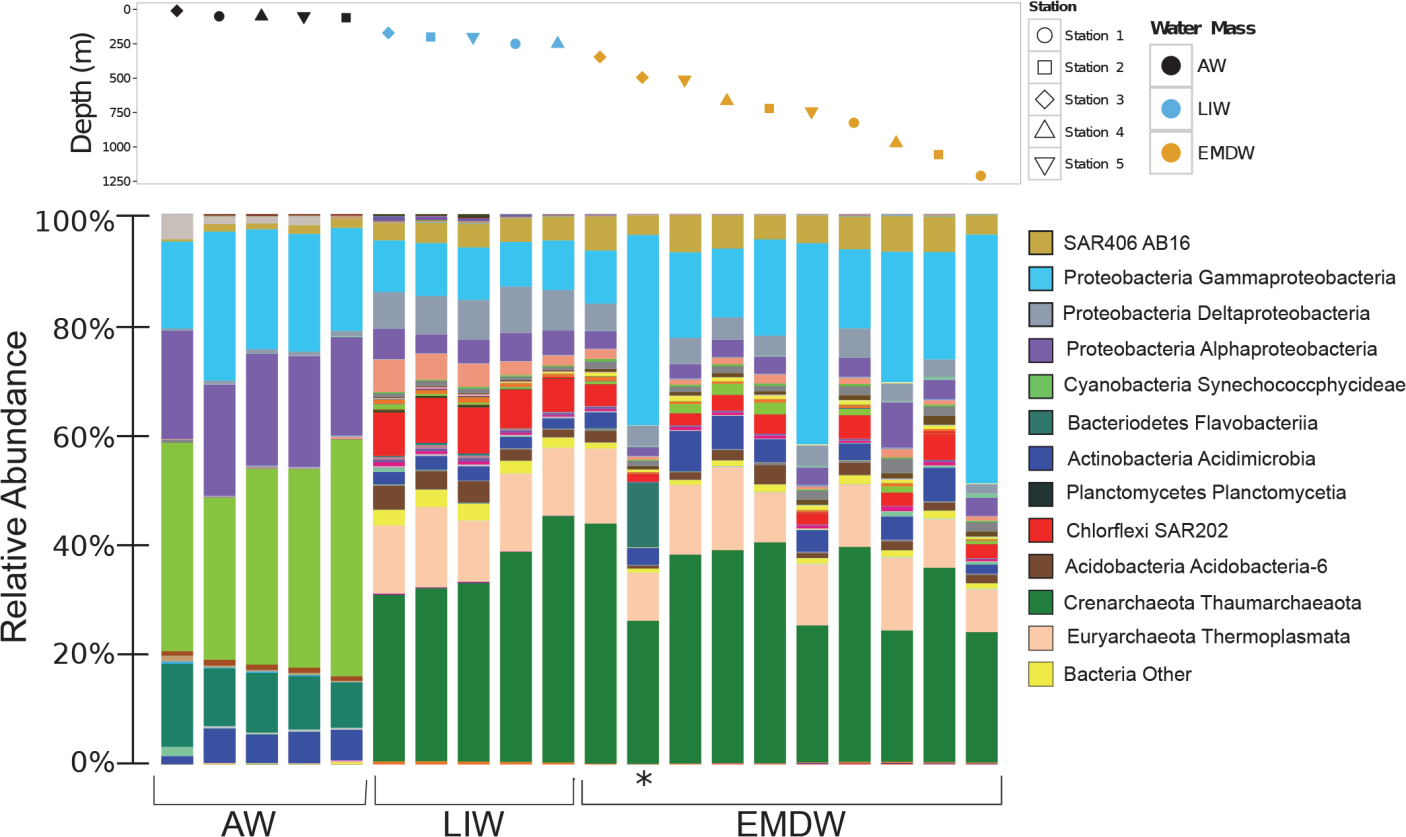
Taxa summaries for samples. Taxa summaries showing the distribution of various classes in each sample. The top panel depicts the depth of each sample. The shape of the point corresponds to sampling station and the color corresponds to the water mass from which that sample was derived. The bottom panel depicts taxa summaries for each sample plotted in order of depth with the shallowest samples on the left of the plot and the deepest samples on the right. Points in the top panel correspond to the taxa bar below the point. The relative abundance of each class in each sample is plotted. The height of each bar represents the percentage that each taxa comprises in that community. The legend depicts only the most abundant classes in these samples. The bar representing the taxa summary for the sample collected directly above the North Alex Mud Volcano is indicated by an asterisk below the taxa summary.

**Fig 5:**
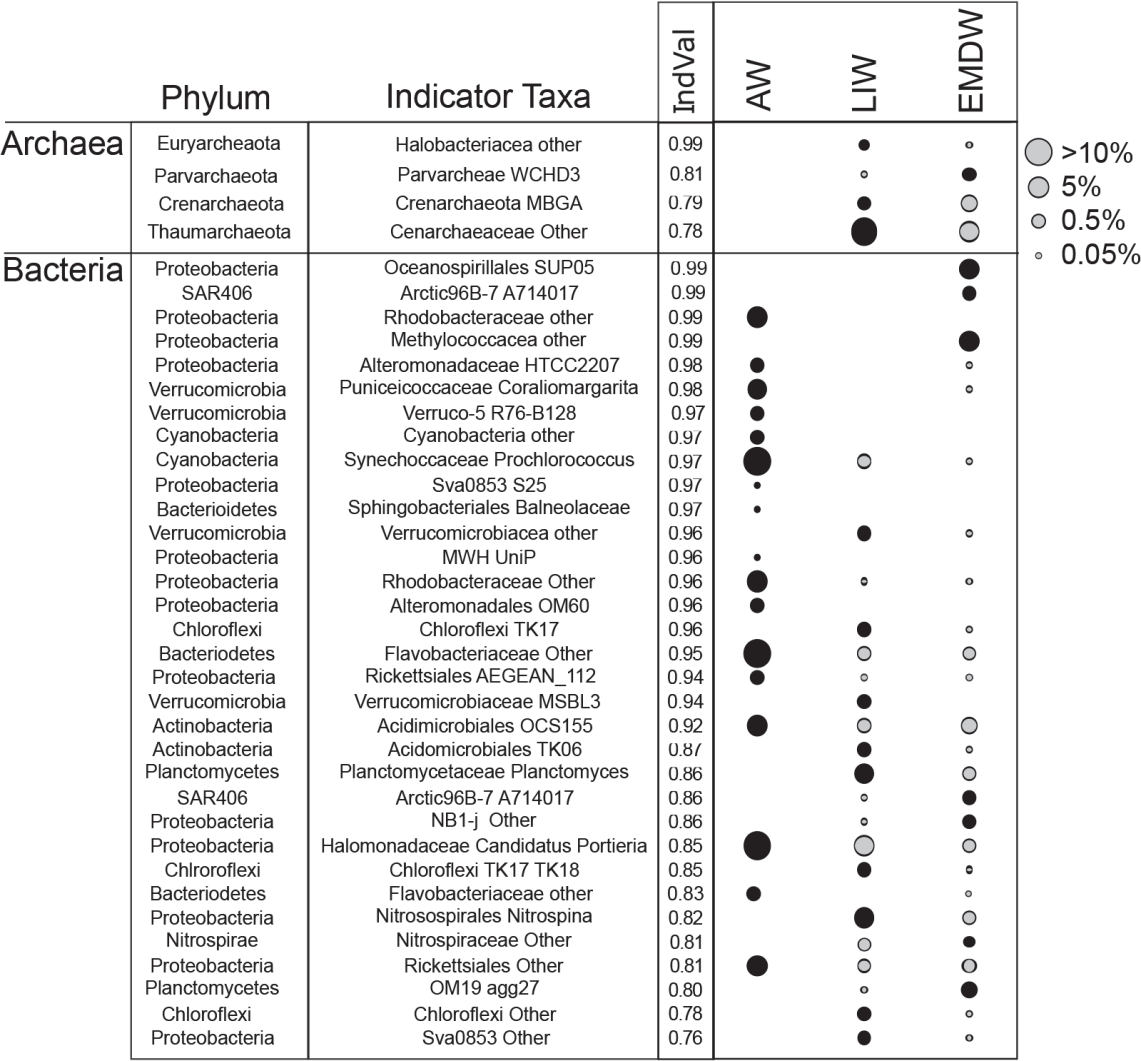
Indicator species analysis. Indicator species of various water masses are shown. Indicator values (IndVal) are shown next to the taxonomic information for the indicator taxa as indicated by IndVal. Size of symbol is proportional to the mean relative abundance in that water mass. Black symbols indicate for which water mass the taxon is an indicator. Gray symbols indicate water masses that contain a taxon, but for which that taxon is not an indicator taxa.

Distinct microbial taxa were found in each water mass (Fig. 4). ANOVA and Tukey HSD tests comparing the relative abundance of microbial classes across water masses revealed that 53 classes show significant differential abundance across the three water masses. Additionally, 37 indicator taxa were identified by indicator species analysis as good indicators of the water masses. The most abundant groups in the AW were *Cyanobacteria*, *Proteobacteria* and *Bacteroidetes*. The *Cyanobacterial* groups in the AW were comprised of both relatives of *Synechococcus* and *Prochlorococcus*. *Synechococcus* were most abundant at the very top of the AW (10 m depth) and *Procholorococcus* dominated the remaining AW samples (50 to 60 m depth). The *Proteobacteria* in the AW were predominantly *Alpha-* and *Gammaproteobacteria*. Furthermore, the dominant *Bacteroidetes* in the AW were primarily from the *Flavobacteria* class. The mean relative abundance of *Flavobacteria* in the AW is 11% of recovered reads, which was ten times greater than the average abundance of *Flavobacteria* in the LIW and EMDW. The abundance of *Alphaproteobacteria* was significantly different between the three water masses (ANOVA p-value 0.0001) with the highest relative abundance of *Alphaproteobacteria* in the AW. Indicator species analysis confirmed this, showing that *Alphaproteobacteria*, as well as *Cyanobacteria*, *Verrucomicrobia*, *Gammaproteobacteria*, and *Bacteriodetes* were all good indicators of the AW (Fig. 5)

*Thaumarchaeota* were the most abundant taxa in the intermediate and deep water mass (LIW and EMDW). Despite the differences in overall microbial community structure between the intermediate and deep-water masses (P_permutated_ = 0.012), the most dominant taxa in the both water masses were *Thaumarchaeota*. *Euryarchaeota* were also a large portion of the recovered reads from the two deeper water masses (8–14% of recovered reads). These *Euryarchaeota* are from the Marine Group II and Marine Group III families.

ANOVA with Tukey HSD test and Indicator Species analysis indicated that *Deltaproteobacteria*, *Planctomycetes*, *Chloroflexi*, *Thaumarchaeota*, and Archaea from the class *Halobacteria* were enriched in and good indicators of the LIW. The relative abundance of *Deltaproteobacteria* was significantly different between the three water masses (ANOVA corrected p-value 2.01E-08) and was higher in the LIW relative to the other two water masses. While, halophilic Archaea were a relatively low percentage of recovered reads in all samples, they are good indicators of the LIW (indicator value: 0.99, q-value: 0.019).

*Gammaproteobacteria* related to *Methylococcales* and *Oceanospirillales*, as well as SAR406 and *Nitrospirae* are indicators of the EMDW, based on indicator species analysis. *Gammaproteobacterial* abundance was significantly different between the three water masses (ANOVA P = 1.43E-07). *Gammaproteobacteria* were present at higher abundance in the EMDW relative to the AW and LIW. Members of the *Parvarchaea* were also good indicators of the EMDW (indicator value: 0.8 q-value: 0.019).

### Analysis of the microbial community directly above the North Alex Mud Volcano

The near-bottom sample from the North Alex Mud Volcano (Station 3) was quite distinct from other samples at similar depths. In particular the relative abundance of *Flavobacteria*, *Methylococcales*, and *Thiotrichales* was higher in this sample relative to other samples from similar depths. (Fig. 4 and S6 Fig.). *Flavobacteria* were present at high relative abundances in the AW, but much lower abundances in the LIW and EMDW. In this mud volcano sample, *Flavobacteria* comprised 12% of recovered reads, which is almost ten times greater than any other deep-water sample. *Thiotrichales* were found at 4% of recovered reads in this samples compared to an average of 0.98% in the rest of the EMDW. Additionally relatives of *Methylococcales* were present at high abundance in this sample (22.7% of recovered reads). *Methylococcales* were only found at similar abundances in samples taken from much deeper depths.

## Discussion

The goal of this study was to identify the effect of physical and geological phenomena on structuring the microbial community in the Eastern Mediterranean water column. Our geochemical analysis demonstrated that the waters of these sites in the Eastern Mediterranean are highly stratified by depth. Chemical and physical stratification at our sampling stations separated according to the three previously described water masses [16]. Our results indicated that higher organic carbon and higher temperatures are characteristics of the AW, the LIW has elevated salinity, and the EMDW has higher nutrient concentrations. Our results also confirm previous reports, which state that phosphate concentrations are low throughout the water column [15, 62]. The distinct physical and chemical properties of these water masses afford distinct niches in which different microbial communities can establish themselves.

Our results also demonstrated that microbial abundance differed by depth. The high cell numbers in the surface waters are in line with previous reports, which demonstrated a decrease in cell numbers with depth [25]. AODC cell counts were an order of magnitude higher than cell counts determined by PLFA analysis. This could be due to the fact that PLFA cell counts are derived from a conversion factor, which is often determined from cultured cells and is also based on bacterial cell size. Therefore, PLFA conversion factors do not always directly relate to cell counts [48]. Additionally, PLFA measurements do not take into account Archaeal lipids. *Thaumarchaeota* are often dominant microbes in deep marine waters [63–66]. Therefore, PLFA biomass estimates from water with high numbers of Archaea, may underestimate microbial cell numbers.

Analysis of PLFA biomarkers confirmed the presence of distinct microbial taxa in each water mass. We have demonstrated that the surface waters have a higher relative mole percent of polyunsaturated lipids. Polyunsaturated lipids are indicative of *Cyanobacteria* (18:2ω6) and Eukaryotes (polyunsaturates with an ω3 double bond) [67]. Our PLFA data suggests that active phototrophic microbes were present at much higher abundance in the surface waters compared to the deeper water masses. Our PLFA results also indicate an increase in mid-branched saturated PLFA in the LIW and EMDW. Mid-branched saturates are biomarkers for metal and sulfate reducing microbes [67], many of which are from the *Deltaproteobacterial* class. The elevated levels of these PLFAs may indicate higher abundance of these microbial taxa in these deep-water masses.

More detailed distinctions in the microbial community structure were observed when analyzing 16S rRNA sequencing data. We demonstrated that microbial diversity (Shannon, Simpson, and phylogenetic diversity) was significantly different between these water masses, with the diversity being highest in the LIW. The lowest diversity was observed in the AW with an increase in diversity in the LIW and a subsequent decrease in the EMDW. Nutrient concentrations gradually increased across the LIW. This gradient may provide a variety of niches capable of supporting diverse communities of microbes in the same water mass. Our results are in line with previous reports that have shown that the diversity of deep-water microbial communities is higher than surface and subsurface waters [11, 14].

Our findings also confirm previous studies, which have shown that microbial communities are distinct in different oceanic water masses [11, 12, 14]. It is not surprising that the microbial community in the photic zone was distinct from that in the aphotic zone, however there were significant and unexpected differences in the microbial community between the LIW and the EMDW. A recent study of the microbial community in the Southwest Atlantic indicated that there were very few differences between deep-water masses in the Southwest Atlantic [14]. However, we observed distinct differences between each of the three water masses in our samples from the Eastern Mediterranean.

*Cyanobacteria* were most abundant in the AW, which has the highest light levels to support growth of phototrophic microbes. The majority of *Cyanobacterial* reads recovered from samples from the AW were related to *Prochlorococcus* species, which are well adapted for growth in nutrient poor surface waters. The stoichiometry required to support growth of *Prochlorococcus* is shifted from the Redfield ratio enabling growth in phosphorus-limited ecosystems such as the Eastern Mediterranean [68, 69]. In addition to *Prochlorococcus*, *Alphaproteobacteria* are significantly enriched in the AW. These *Alphaproteobacteria* are related to *Rhodosprillales*, *Rhodobacteriales*, *and Rickettsiales*, which are related to SAR-11—a ubiquitous bacterium in marine surface waters [70]. Both *Prochlorococcus* species and members of the *Alphaproteobacteria* are well adapted for growth in oligotrophic environments [71]. The nutrient concentrations in the AW are much lower than in other water masses and in many cases below detection. These low nutrient concentrations may select for these microbes, which are capable of thriving under nutrient limitation. Members of the *Flavobacteria* were also found at significantly higher levels in the AW relative to the deeper two water masses. Members of the *Flavobacteria* have been shown to be involved in degradation of high molecular weight organic matter [72] and could be involved in degrading the organic matter that is produced by phototrophic *Cyanobacteria* and algae in the AW.

*Thaumarchaeota* related to cosmopolitan ammonia oxidizing Archaea [63–66] were dominant members of the deep-water community representing between 18–40% of the recovered reads for samples from the LIW and EMDW. The fact that *Thaumarchaeota* dominated samples from the LIW and EMDW indicates that they perform an essential function in the intermediate and deep-water environments of the Eastern Mediterranean and may be important players in the nitrogen cycle of the Mediterranean deep-water. Elevated levels of *Deltaproteobacteria* were also a defining factor of the LIW. Many of these *Deltaproteobacteria* are closely related to SAR324, which are physiologically diverse group [73]. Some microbes in the SAR324 group have the ability to grow autotrophically and heterotrophically participating in carbon, sulfur and nitrogen cycling [73, 74]. Their presence at elevated levels in the LIW may indicate that some of these processes are occurring in this intermediate water mass.

Some of the important taxa in the LIW are putatively involved in nitrogen cycling. For example, *Nitrospinaceae*, *Planctomycetes* and *Chloroflexi* from the SAR202 class are all important member of the microbial community in the LIW. *Nitrospinaceae* related to the autotrophic nitrite oxidizing bacterium, *Nitrospina*, are good indicators of the LIW (indicator value 0.82 q-value 0.019). Members of the *Planctomycetes* are quite diverse in their ecology and functional capacity, with some *Planctomycetes* able to perform anaerobic ammonia oxidation (annamox), which is an essential process in the nitrogen cycle of many marine environments [75],[76]. *Chloroflexi* from the SAR202 class were also found at high abundances in the LIW (10–15% of recovered reads). It has been proposed that Members of the SAR202 class are involved in amino acid mineralization [77]. These findings indicate that some of the microbes in the LIW are putatively involved in autotrophic and heterotrophic processes related to nitrogen cycling. The importance of nitrogen cycling in the Mediterranean deep-water was suggested by a recent study which showed that nitrogen fixation in the aphotic zone of the Eastern Mediterranean was quite high [24]. Our finding that the predominant taxa in the LIW were related to microbes involved in nitrogen cycling processes suggests that the LIW is an essential part of the nitrogen cycle of the Mediterranean Sea.

We also found organisms that were indicative of the different water masses. Our results demonstrate that the LIW has the highest salinity of the three water masses in the Eastern Mediterranean. Correspondingly, we found halophilic Archaea from the class *Halobacteria* were good indicators of the LIW (indicator value 0.99, q value 0.019). This high indicator value suggests that *Halobacteria* were found in most of the samples from LIW and were absent in lower salinity samples from the AW and EMDW. The ability of *Halobacteria* to thrive under high salt conditions [78] may allow them to colonize the higher salinity LIW.

*Parvarchaeota* were indicators of the EMDW. The *Parvarchaeota* are a poorly understood group in a newly described super phyla unified by their small cell size and small genome size [79, 80]. Their role in the EMDW microbial community is still unclear, and requires further investigation. *Gammaproteobacteria* from the *Oceanospirillales* and *Methylococcales* orders were present at high abundance in the EMDW microbial community. The most abundant *Oceanospirillales* in the EMDW were most closely related to the SUP05 and *Halomonadaceae* families. Relatives of SUP05 have been found as a part of sulfur oxidizing assemblages in different environments [81, 82]. There is metagenomic and physiological evidence that the SUP05 group is involved in autotrophic growth involving sulfur and hydrogen oxidation. Microbes related to SUP05 were indicators of the EMDW (indicator value of 0.99, q-value of 0.019) suggesting that sulfur and hydrogen oxidation may be occurring in the EMDW.

Additionally, Sequences corresponding to *Methylococcales* comprised between 5–20% of the recovered reads in samples from the EMDW and were indicator species for the EMDW (Indicator value 0.99, q-value 0.019). *Methylococcales* are methanotrophs and typically acquire their carbon and energy from methane oxidation. The dominance of sequences related to known methanotrophs suggests that methane oxidation may be another important physiology in the deep waters of the Eastern Mediterranean. Many of the microbes in the deeper water masses are putatively involved in autotrophic physiologies. Therefore, it is possible that the oligotrophy of the Eastern Mediterranean has selected for a set of autotrophic microbes that use the available nutrients in energy generating processes.

Another goal of this study was to characterize the effect of geological phenomena such as mud volcanism on the microbial community of the water column. Our results demonstrate that the microbial community directly above the North Alex Mud Volcano was distinct from the microbial community in other samples from similar depths. However, the microbial community in the upper water column (i.e. AW and LIW) above the North Alex Mud Volcano resembled the microbial community from samples collected within the same water masses in other sampling locations. Members of the *Flavobacteriales*, *Thiotrichales* and *Methylococcales* were present at higher abundances in this mud volcano sample as compared to samples from similar depths from other stations. *Flavobacteria* have been implicated in degradation of high molecular weight organic matter [72]. It may be that the mud volcano has enriched for microbes capable of utilizing the emitted organic material from the mud volcano. The *Thiotrichales* sequences in this sample are most similar to members of family *Piscirickettsiaceae*. PAH-degrading *Cycloclasticus* and halophilic methylotrophic *Methylophaga* species are members of the *Piscirickettsiaceae*. *Methylococcales* are present at much higher levels in this sample than in samples from similar depths. The elevated abundance of *Thiotrichales* and *Methylococcales* in this samples could be indicative of active release of methane and other hydrocarbon compounds into the water column above the North Alex Mud Volcano.

The increased abundance of known hydrocarbon degraders as well as a distinct community structure directly above the mud volcano suggests that mud volcanism affects the microbial community in the overlaying waters. Further genomic work is required to better understand the role of these and *Flavobacteria* and other putative hydrocarbon degrading microbes in the deep-water community, especially in the water column adjacent to natural seeps.

## Conclusion

This study employed PLFA analysis and massively parallel 16S rRNA gene sequencing to determine effect of water stratification and mud volcanism on the microbial community of the Eastern Mediterranean water column. The adjacent water masses of the Mediterranean have selected for distinct microbial communities able to colonize these separate water masses. Our results contribute to the growing body of work, which demonstrates that physical factors in addition to geochemical parameters influence microbial community structure. Our results also clarify importance of the deep-water microbial communities of the Eastern Mediterranean in nitrogen cycling. In particular, the microbial community in the LIW contains a number of microbes putatively involved in nitrogen cycling and may be essential to the nitrogen cycle of the Eastern Mediterranean.

Our study also demonstrated that geological phenomena such as mud volcanism drastically affect the microbial communities present in the water column by enriching for microbes known to utilize hydrocarbons and high molecular weight organic matter. These data combine to underscore the important role that physical, geological, and geochemical factors play in shaping marine microbial communities.

## Supporting Information

S1 Fig Temperature and Salinity plots. Temperature and salinity profiles for stations two three, four, and five. Temperature is shown in red and salinity is shown in green. (A) Station 2, (B) Station 3, (C) Station 4, (D) Station 5.
(TIF)

S2 Fig Nutrients and NPOC by depth. (A) Total Nitrogen (B) Nitrate (C) Inorganic Phosphate (D) Ammonia (E) NPOC (F) Silicate (G) Sulfate (H) Iron. Shapes represent different sampling stations. Colors represent water masses.
(TIF)

S3 Fig Alpha diversity. (A) Shannon diversity plotted as a function of depth. Symbols represent sampling location and colors represent water masses. (B) Shannon diversity for each water mass. Symbols represent sampling locations. Colors correspond to depth. (C) PD Whole Tree diversity plotted as a function of depth. Symbols represent sampling location and colors represent water masses. (D) PD Whole Tree diversity for each water mass. Symbols represent sampling locations. Colors correspond to depth. (E) Simpson diversity plotted as a function of depth. Symbols represent sampling location and colors represent water masses. (F) Simpson diversity for each water mass. Symbols represent sampling locations. Colors correspond to depth.
(TIF)

S4 Fig Beta Diversity by Depth. (A) Hierarchichal Clustering Analysis. Symbols represent sampling station and colors correspond to depth. (B)Non-metric multidimensional scaling analysis of weighted unifrac distances. Geochemical variable were fit to the weighted unifrac distance matric. Vectors are shown for environmental variables that fit the data with a p value of greater than 0.01. Shapes represent sampling station. Colors correspond to depth.
(TIF)

S5 Fig NMDS plots of Archaea and Bacteria. (A and B) NMDS plot of weighted unifrac distances determined for an OTU table of OTUs classified as Archaea. (A) Symbols represent sampling station and colors represent water mass (B) symbols represent sampling station colors correspond to depth. (C and D) NMDS plot of weighted unifrac distances determined for an OTU table of OTUs classified as Bacteria. (C) Symbols represent sampling station and colors represent water mass (D) symbols represent sampling station colors correspond to depth.
(TIF)

S6 Fig Microbial Classes Enriched in the Sample Directly Above the North Alex Mud Volcano. Three microbial classes were highly enriched in the water sample directly above the North Alex Mud Volcano. (A) Relative abundance of *Flavobacteriales* plotted as a function of depth. Symbols represent sampling location. Colors represent water mass. (B) Relative abundance of *Methylococcales* plotted as a function of depth. Symbols represent sampling location. Colors represent water mass. (C) Relative abundance of *Thiotrichales* plotted as a function of depth. Symbols represent sampling location. Colors represent water mass.
(TIF)

S1 Table Geochemical data for each sample: Physical, chemical and nutrient data for each of the 20 samples collected. (DOCX)

S2 Table PLFA Data. Mole percent for each lipid detected in each samples. Groups of samples are shown according to the water mass from which they were obtained. In the sample names, the first number corresponds to the sampling station and the second number is the unique identifier for that sample. The average of each lipid in each water mass is shown as well as the minimum and maximum mole percent values for each lipid in each water mass.
(DOCX)

S3 Table Number of sequences per sample: Number of 16S rRNA sequences for each sample after quality filtering and chimera checking. The sample station, depth and water mass for each sample are also shown for each sample.
(DOCX)

S4 Table Microbial classes significantly different between the three water masses. Two-way ANOVA and Tukey tests were used to compare the relative abundance of microbial classes in order to determine which classes were significantly different between the three water masses. ANOVA F statistic and p value are shown for each taxa. p values were corrected using the false discovery rate calculation in R. Tukey test was used to determine which water masses were significantly different from each other for that class. The mean relative abundance of each class is also reported for each water mass.
(DOCX)

S5 Table Indicator species values for all indicator taxa: Indicator taxa with their indicator species values and their corrected Pvalues as well as the water mass for which they are indicators. (DOCX)

## References

[1] Brockett BFT, Prescott CE, Grayston SJ. Soil moisture is the major factor influencing microbial community structure and enzyme activities across seven biogeoclimatic zones in western Canada. Soil Biol Biochem. 2012;44(1):9–20.

[2] Dang HY, Chen RP, Wang L, Guo LZ, Chen PP, Tang ZW, et al Environmental Factors Shape Sediment Anammox Bacterial Communities in Hypernutrified Jiaozhou Bay, China. Applied and Environmental Microbiology. 2010;76(21):7036–47. 10.1128/AEM.01264-10 20833786 PMC2976235

[3] Steenbergh AK, Bodelier PLE, Slomp CP, Laanbroek HJ. Effect of Redox Conditions on Bacterial Community Structure in Baltic Sea Sediments with Contrasting Phosphorus Fluxes (vol 9, e92401, 2014). Plos One. 2014;9(7). 10.1371/journal.pone.0092401 PMC3965429 24667801

[4] Fagervold SK, Bourgeois S, Pruski AM, Charles F, Kerherve P, Vetion G, et al River organic matter shapes microbial communities in the sediment of the Rhone prodelta. Isme J. 2014;8(11):2327–38. 10.1038/ismej.2014.86 24858780 PMC4992087

[5] Adams HE, Crump BC, Kling GW. Temperature controls on aquatic bacterial production and community dynamics in arctic lakes and streams. Environ Microbiol. 2010;12(5):1319–33. 10.1111/j.1462-2920.2010.02176.x 20192972

[6] Moore CM, Mills MM, Arrigo KR, Berman-Frank I, Bopp L, Boyd PW, et al Processes and patterns of oceanic nutrient limitation. Nat Geosci. 2013;6(9):701–10.

[7] Urakawa H, Martens-Habbena W, Huguet C, de la Torre JR, Ingalls AE, Devol AH, et al Ammonia availability shapes the seasonal distribution and activity of archaeal and bacterial ammonia oxidizers in the Puget Sound Estuary. Limnol Oceanogr. 2014;59(4):1321–35.

[8] Wilkins D, van Sebille E, Rintoul SR, Lauro FM, Cavicchioli R. Advection shapes Southern Ocean microbial assemblages independent of distance and environment effects. Nature communications. 2013;4:2457 10.1038/ncomms3457 24036630

[9] Hamdan LJ, Coffin RB, Sikaroodi M, Greinert J, Treude T, Gillevet PM. Ocean currents shape the microbiome of Arctic marine sediments. Isme J. 2013;7(4):685–96. 10.1038/ismej.2012.143 23190727 PMC3603395

[10] Bouman HA, Ulloa O, Barlow R, Li WK, Platt T, Zwirglmaier K, et al Water-column stratification governs the community structure of subtropical marine picophytoplankton. Environ Microbiol Rep. 2011;3(4):473–82. 10.1111/j.1758-2229.2011.00241.x 23761310

[11] Agogue H, Lamy D, Neal PR, Sogin ML, Herndl GJ. Water mass-specificity of bacterial communities in the North Atlantic revealed by massively parallel sequencing. Mol Ecol. 2011;20(2):258–74. 10.1111/j.1365-294X.2010.04932.x 21143328 PMC3057482

[12] DeLong EF, Preston CM, Mincer T, Rich V, Hallam SJ, Frigaard NU, et al Community genomics among stratified microbial assemblages in the ocean's interior. Science. 2006;311(5760):496–503. 16439655 10.1126/science.1120250

[13] Seymour JR, Doblin MA, Jeffries TC, Brown MV, Newton K, Ralph PJ, et al Contrasting microbial assemblages in adjacent water masses associated with the East Australian Current. Env Microbiol Rep. 2012;4(5):548–55. 23760900 10.1111/j.1758-2229.2012.00362.x

[14] Alves Junior N, Meirelles P, de Oliveira Santos E, Dutilh B, Silva GZ, Paranhos R, et al Microbial community diversity and physical–chemical features of the Southwestern Atlantic Ocean. Archives of microbiology. 2014;10:1–15. 10.1007/s00203-014-1035-6 25205422

[15] Krom MD, Emeis KC, Van Cappellen P. Why is the Eastern Mediterranean phosphorus limited? Prog Oceanogr. 2010;85(3–4):236–44.

[16] Skliris N. Past, Present and Future Patterns of the Thermohaline Circulation and Characteristic Water Masses of the Mediterranean Sea In: Goffredo S, Dubinsky Z, editors. The Mediterranean Sea. Springer Netherlands; 2014 p. 29–48.

[17] Mella-Flores D, Mazard S, Humily F, Partensky F, Mahe F, Bariat L, et al Is the distribution of Prochlorococcus and Synechococcus ecotypes in the Mediterranean Sea affected by global warming? Biogeosciences. 2011;8(9):2785–804.

[18] Lamy D, Jeanthon C, Cottrell MT, Kirchman DL, Van Wambeke F, Ras J, et al Ecology of aerobic anoxygenic phototrophic bacteria along an oligotrophic gradient in the Mediterranean Sea. Biogeosciences. 2011;8(4):973–85.

[19] Feingersch R, Suzuki MT, Shmoish M, Sharon I, Sabehi G, Partensky F, et al Microbial community genomics in eastern Mediterranean Sea surface waters. Isme J. 2010;4(1):78–87. 10.1038/ismej.2009.92 19693100

[20] Pantoja S, Repeta DJ, Sachs JP, Sigman DM. Stable isotope constraints on the nitrogen cycle of the Mediterranean Sea water column. Deep-Sea Res Pt I. 2002;49(9):1609–21.

[21] Bethoux JP, Copinmontegut G. Biological Fixation of Atmospheric Nitrogen in the Mediterranean-Sea. Limnol Oceanogr. 1986;31(6):1353–8.

[22] Ibello V, Cantoni C, Cozzi S, Civitarese G. First basin-wide experimental results on N-2 fixation in the open Mediterranean Sea. Geophys Res Lett. 2010;37.

[23] Yogev T, Rahav E, Bar-Zeev E, Man-Aharonovich D, Stambler N, Kress N, et al Is dinitrogen fixation significant in the Levantine Basin, East Mediterranean Sea? Environ Microbiol. 2011;13(4):854–71. 10.1111/j.1462-2920.2010.02402.x 21244595

[24] Rahav E, Bar-Zeev E, Ohayon S, Elifantz H, Belkin N, Herut B, et al Dinitrogen fixation in aphotic oxygenated marine environments. Front Microbiol. 2013;4. 10.3389/fmicb.2013.00227 PMC3753716 23986748

[25] Yokokawa T, De Corte D, Sintes E, Herndl GJ. Spatial patterns of bacterial abundance, activity and community composition in relation to water masses in the eastern Mediterranean Sea. Aquat Microb Ecol. 2010;59(2):185–95.

[26] Felden J, Lichtschlag A, Wenzhofer F, de Beer D, Feseker T, Ristova PP, et al Limitations of microbial hydrocarbon degradation at the Amon mud volcano (Nile deep-sea fan). Biogeosciences. 2013;10(5):3269–83.

[27] Heijs SK, Laverman AM, Forney LJ, Hardoim PR, van Elsas JD. Comparison of deep-sea sediment microbial communities in the Eastern Mediterranean. Fems Microbiol Ecol. 2008;64(3):362–77. 10.1111/j.1574-6941.2008.00463.x 18422633

[28] Mastalerz V, de Lange GJ, Dahlmann A. Differential aerobic and anaerobic oxidation of hydrocarbon gases discharged at mud volcanoes in the Nile deep-sea fan. Geochim Cosmochim Ac. 2009;73(13):3849–63.

[29] Omoregie EO, Niemann H, Mastalerz V, de Lange GJ, Stadnitskaia A, Mascle J, et al Microbial methane oxidation and sulfate reduction at cold seeps of the deep Eastern Mediterranean Sea. Mar Geol. 2009;261(1–4):114–27.

[30] Loncke L, Gaullier V, Mascle J, Vendeville B, Camera L. The Nile deep-sea fan: An example of interacting sedimentation, salt tectonics, and inherited subsalt paleotopographic features. Mar Petrol Geol. 2006;23(3):297–315.

[31] Dupre S, Woodside J, Klaucke I, Mascle J, Foucher JP. Widespread active seepage activity on the Nile Deep Sea Fan (offshore Egypt) revealed by high-definition geophysical imagery. Mar Geol. 2010;275(1–4):1–19.

[32] Loncke L, Mascle J, Parties FS. Mud volcanoes, gas chimneys, pockmarks and mounds in the Nile deep-sea fan (Eastern Mediterranean): geophysical evidences. Mar Petrol Geol. 2004 21(6):669–89.

[33] Ritt B, Pierre C, Gauthier O, Wenzhofer F, Boetius A, Sarrazin J. Diversity and distribution of cold-seep fauna associated with different geological and environmental settings at mud volcanoes and pockmarks of the Nile Deep-Sea Fan. Mar Biol. 2011;158(6):1187–210.

[34] Mills CT, Dias RF, Graham D, Mandernack KW. Determination of phospholipid fatty acid structures and stable carbon isotope compositions of deep-sea sediments of the Northwest Pacific, ODP site 1179. Mar Chem. 2006;98(2):197–209.

[35] White DC, Pinkart HC, Ringelberg DB. Biomass measurements: Biochemical approaches In: Hurst CH, Knudsen G, McInerney M, Stetzenach LD, Walter M, editors. Manual of Environmental Microbiology. Washington, DC: American Society for Microbiology Press; 1996 p. 91–101.

[36] Hazen TC, Rocha AM, Techtmann SM. Advances in monitoring environmental microbes. Curr Opin Biotech. 2013;24(3):526–33. 10.1016/j.copbio.2012.10.020 23183250

[37] Kelley D. oce: Analysis of Oceanographic data. R package version 09–12 Available: http://cran.r-project.org/web/packages/oce/. Accessed 11 February 2015.

[38] Schlitzer R. Ocean Data View. Available: http://odv.awi.de/. 2014. Accessed 11 February 2015.

[39] R Core Team. R: A Language and Environment for Statistical Computing. R Foundation for Statistical Computing Vienna, Austria Available: http://www.r-project.org/. Accessed 11 February 2015.

[40] Kerouel R, Aminot A. Fluorometric determination of ammonia in sea and estuarine waters by direct segmented flow analysis. Mar Chem. 1997;57(3–4):265–75.

[41] Armstron FAJ, Stearns CR, Strickland JDH. Measurement of Upwelling and Subsequent Biological Processes by Means of Technicon Autoanalyzer and Associated Equipment. Deep-Sea Res. 1967;14(3):381–389.

[42] Grasshoff K, Ehrhardt M, Kremling K. Methods of Seawater Analysis1983. Basel: Verlag Chimie; 1983.

[43] Murphy J, Riley JP. A Modified Single Solution Method for Determination of Phosphate in Natural Waters. Anal Chim Acta. 1962;26(1):31.

[44] Anderson MJ. A new method for non-parametric multivariate analysis of variance. Austral Ecol. 2001;26(1):32–46.

[45] Oksanen J, Blanchet FG, Kindt R, Legendre P, Minchin PR, O'Hara RB, et al. vegan: Community Ecology Package. R package version 20–10. Available: http://cran.r-project.org/web/packages/vegan/. Accessed 11 February 2015.

[46] Francisco DE, Mah RA, Rabin AC. Acridine Orange-Epifluorescence Technique for Counting Bacteria in Natural Waters. T Am Microsc Soc. 1973;92(3):416–21. 4581469

[47] White DC, Ringelberg DB. Signature lipid biomarker analysis In: Burlage RS, Atlas R, Stahl DA, Geesey G, Sayler GS, editors. Techniques in microbial ecology. New York: Oxford University Press; 1998.

[48] Green CT, Scow KM. Analysis of phospholipid fatty acids (PLFA) to characterize microbial communities in aquifers. Hydrogeol J. 2000;8(1):126–41.

[49] Hazen TC, Dubinsky EA, DeSantis TZ, Andersen GL, Piceno YM, Singh N, et al Deep-Sea Oil Plume Enriches Indigenous Oil-Degrading Bacteria. Science. 2010;330(6001):204–8. 10.1126/science.1195979 20736401

[50] Caporaso JG, Lauber CL, Walters WA, Berg-Lyons D, Huntley J, Fierer N, et al Ultra-high-throughput microbial community analysis on the Illumina HiSeq and MiSeq platforms. Isme J. 2012;6(8):1621–4. 10.1038/ismej.2012.8 22402401 PMC3400413

[51] Caporaso JG, Kuczynski J, Stombaugh J, Bittinger K, Bushman FD, Costello EK, et al QIIME allows analysis of high-throughput community sequencing data. Nat Methods. 2010;7(5):335–6. 10.1038/nmeth.f.303 20383131 PMC3156573

[52] Aronesty E. ea-utils: "Command-line tools for processing biological sequencing data". Available: https://code.google.com/p/ea-utils/. Accessed 11 February 2015.

[53] Edgar RC. Search and clustering orders of magnitude faster than BLAST. Bioinformatics. 2010;26(19):2460–1. 10.1093/bioinformatics/btq461 20709691

[54] Edgar RC, Haas BJ, Clemente JC, Quince C, Knight R. UCHIME improves sensitivity and speed of chimera detection. Bioinformatics. 2011;27(16):2194–200. 10.1093/bioinformatics/btr381 21700674 PMC3150044

[55] Caporaso JG, Bittinger K, Bushman FD, DeSantis TZ, Andersen GL, Knight R. PyNAST: a flexible tool for aligning sequences to a template alignment. Bioinformatics. 2010;26(2):266–7. 10.1093/bioinformatics/btp636 19914921 PMC2804299

[56] Wang Q, Garrity GM, Tiedje JM, Cole JR. Naive Bayesian classifier for rapid assignment of rRNA sequences into the new bacterial taxonomy. Applied and Environmental Microbiology. 2007;73(16):5261–7. 17586664 10.1128/AEM.00062-07 PMC1950982

[57] Bray JR, Curtis JT. An Ordination of the Upland Forest Communities of Southern Wisconsin. Ecol Monogr. 1957;27(4):326–49.

[58] Lozupone C, Knight R. UniFrac: a new phylogenetic method for comparing microbial communities. Applied and Environmental Microbiology. 2005;71(12):8228–35. 16332807 10.1128/AEM.71.12.8228-8235.2005 PMC1317376

[59] Goslee SC, Urban DL. The ecodist package for dissimilarity-based analysis of ecological data. J Stat Softw. 2007;22(7):1–19.

[60] Roberts DW. labdsv: Ordination and Multivariate Analysis for Ecology. R package version 16-1. Available: http://cran.r-project.org/web/packages/labdsv. Accessed 11 February 2015.

[61] Dufrene M, Legendre P. Species assemblages and indicator species: The need for a flexible asymmetrical approach. Ecol Monogr. 1997;67(3):345–66.

[62] Thingstad TF, Krom MD, Mantoura RFC, Flaten GAF, Groom S, Herut B, et al Nature of Phosphorus Limitation in the Ultraoligotrophic Eastern Mediterranean. Science. 2005;309(5737):1068–71. 16099984 10.1126/science.1112632

[63] Konneke M, Bernhard AE, de la Torre JR, Walker CB, Waterbury JB, Stahl DA. Isolation of an autotrophic ammonia-oxidizing marine archaeon. Nature. 2005;437(7058):543–6. 16177789 10.1038/nature03911

[64] Walker CB, de la Torre JR, Klotz MG, Urakawa H, Pinel N, Arp DJ, et al Nitrosopumilus maritimus genome reveals unique mechanisms for nitrification and autotrophy in globally distributed marine crenarchaea. P Natl Acad Sci USA. 2010;107(19):8818–23. 10.1073/pnas.0913533107 20421470 PMC2889351

[65] Fuhrman JA, Mccallum K, Davis AA. Novel Major Archaebacterial Group from Marine Plankton. Nature. 1992;356(6365):148–9. 1545865 10.1038/356148a0

[66] Delong EF. Archaea in Coastal Marine Environments. P Natl Acad Sci USA. 1992 Jun 15;89(12):5685–9. 1608980 10.1073/pnas.89.12.5685 PMC49357

[67] Findlay RH, Dobbs FC. Quantitative description of microbial communities using lipid analysis. Handbook of methods in aquatic microbial ecology. 1993:271–84.

[68] Krumhardt KM, Callnan K, Roache-Johnson K, Swett T, Robinson D, Reistetter EN, et al Effects of phosphorus starvation versus limitation on the marine cyanobacterium Prochlorococcus MED4 I: uptake physiology. Environ Microbiol. 2013;15(7):2114–28. 10.1111/1462-2920.12079 23387819

[69] Reistetter EN, Krumhardt K, Callnan K, Roache-Johnson K, Saunders JK, Moore LR, et al Effects of phosphorus starvation versus limitation on the marine cyanobacterium Prochlorococcus MED4 II: gene expression. Environ Microbiol. 2013;15(7):2129–43. 10.1111/1462-2920.12129 23647921

[70] Morris RM, Rappe MS, Connon SA, Vergin KL, Siebold WA, Carlson CA, et al SAR11 clade dominates ocean surface bacterioplankton communities. Nature. 2002;420(6917):806–10. 12490947 10.1038/nature01240

[71] Sowell SM, Wilhelm LJ, Norbeck AD, Lipton MS, Nicora CD, Barofsky DF, et al Transport functions dominate the SAR11 metaproteome at low-nutrient extremes in the Sargasso Sea. Isme J. 2009;3(1):93–105. 10.1038/ismej.2008.83 18769456

[72] Fernandez-Gomez B, Richter M, Schuler M, Pinhassi J, Acinas SG, Gonzalez JM, et al Ecology of marine Bacteroidetes: a comparative genomics approach. Isme J. 2013;7(5):1026–37. 10.1038/ismej.2012.169 23303374 PMC3635232

[73] Sheik CS, Jain S, Dick GJ. Metabolic flexibility of enigmatic SAR324 revealed through metagenomics and metatranscriptomics. Environ Microbiol. 2014;16(1):304–17. 10.1111/1462-2920.12165 23809230

[74] Swan BK, Martinez-Garcia M, Preston CM, Sczyrba A, Woyke T, Lamy D, et al Potential for Chemolithoautotrophy Among Ubiquitous Bacteria Lineages in the Dark Ocean. Science. 2011;333(6047):1296–300. 10.1126/science.1203690 21885783

[75] Fuerst JA, Sagulenko E. Beyond the bacterium: planctomycetes challenge our concepts of microbial structure and function. Nat Rev Microbiol. 2011;9(6):403–13. 10.1038/nrmicro2578 21572457

[76] Dalsgaard T, Thamdrup B, Canfield DE. Anaerobic ammonium oxidation (anammox) in the marine environment. Res Microbiol. 2005;156(4):457–64. 15862442 10.1016/j.resmic.2005.01.011

[77] Varela MM, van Aken HM, Herndl GJ. Abundance and activity of Chloroflexi-type SAR202 bacterioplankton in the meso- and bathypelagic waters of the (sub)tropical Atlantic. Environ Microbiol. 2008;10(7):1903–11. 10.1111/j.1462-2920.2008.01627.x 18422640

[78] Oren A. Microbial life at high salt concentrations: phylogenetic and metabolic diversity. Saline systems. 2008;4:2 10.1186/1746-1448-4-2 18412960 PMC2329653

[79] Rinke C, Schwientek P, Sczyrba A, Ivanova NN, Anderson IJ, Cheng JF, et al Insights into the phylogeny and coding potential of microbial dark matter. Nature. 2013;499(7459):431–7. 10.1038/nature12352 23851394

[80] Baker BJ, Comolli LR, Dick GJ, Hauser LJ, Hyatt D, Dill BD, et al Enigmatic, ultrasmall, uncultivated Archaea. P Natl Acad Sci USA. 2010;107(19):8806–11. 10.1073/pnas.0914470107 20421484 PMC2889320

[81] Anantharaman K, Breier JA, Sheik CS, Dick GJ. Evidence for hydrogen oxidation and metabolic plasticity in widespread deep-sea sulfur-oxidizing bacteria. P Natl Acad Sci USA. 2013;110(1):330–5. 10.1073/pnas.1215340110 23263870 PMC3538260

[82] Glaubitz S, Kiesslich K, Meeske C, Labrenz M, Jurgens K. SUP05 Dominates the Gammaproteobacterial Sulfur Oxidizer Assemblages in Pelagic Redoxclines of the Central Baltic and Black Seas. Applied and Environmental Microbiology. 2013;79(8):2767–76. 10.1128/AEM.03777-12 23417000 PMC3623164

