# Supplementary figures and images for: The Unique Chemistry of Eastern Mediterranean Water Masses Selects for Distinct Microbial Communities by Depth
Source: PLoS One. 2015 Mar 25;10(3):e0120605. doi: 10.1371/journal.pone.0120605 (PMC4373936; doi:10.1371/journal.pone.0120605)

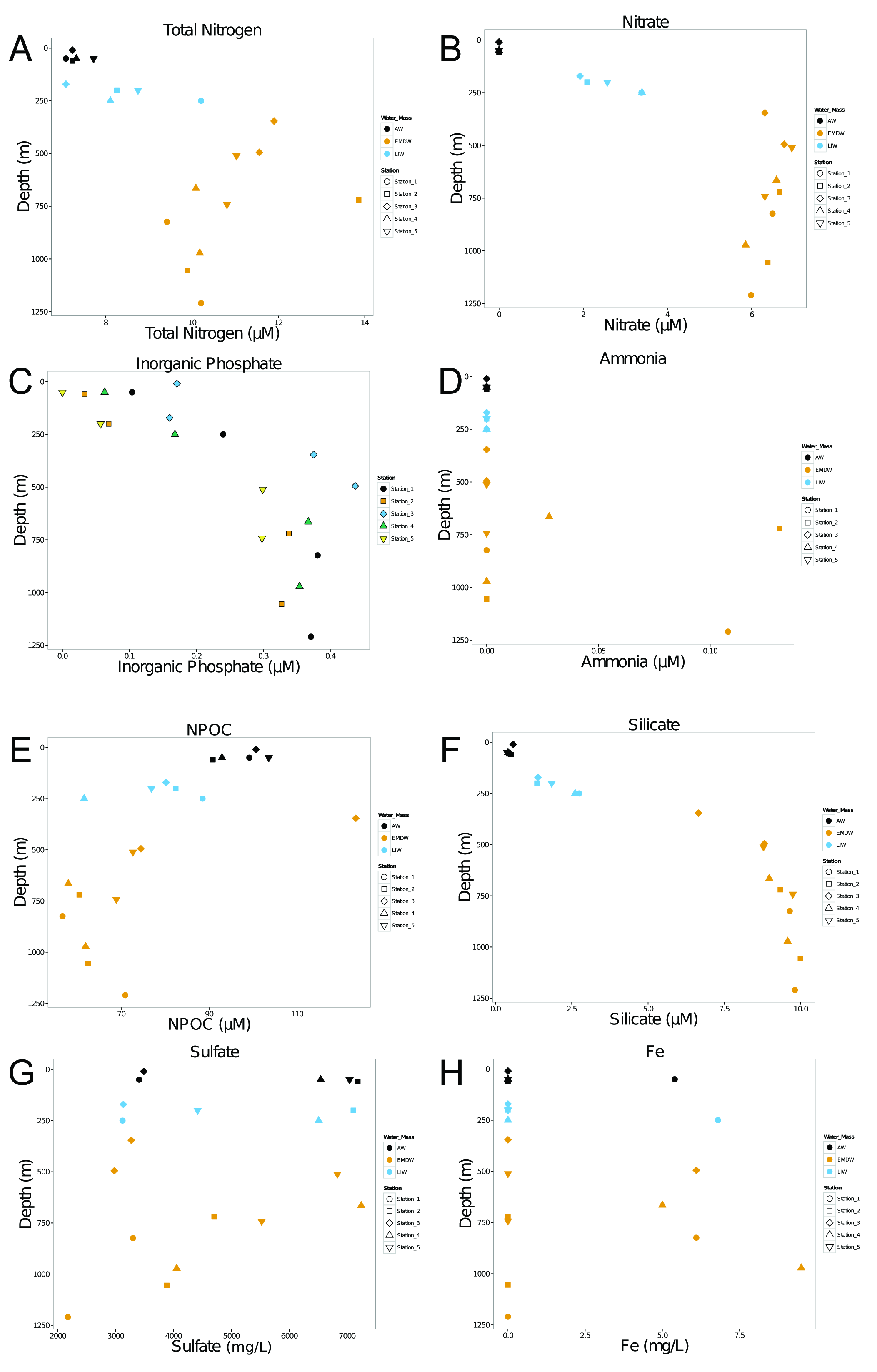

Supplement: S1 Fig — Temperature and salinity profiles for stations two three, four, and five. Temperature is shown in red and salinity is shown in green. (A) Station 2, (B) Station 3, (C) Station 4, (D) Station 5. (TIF) [file pone.0120605.s001.tif]

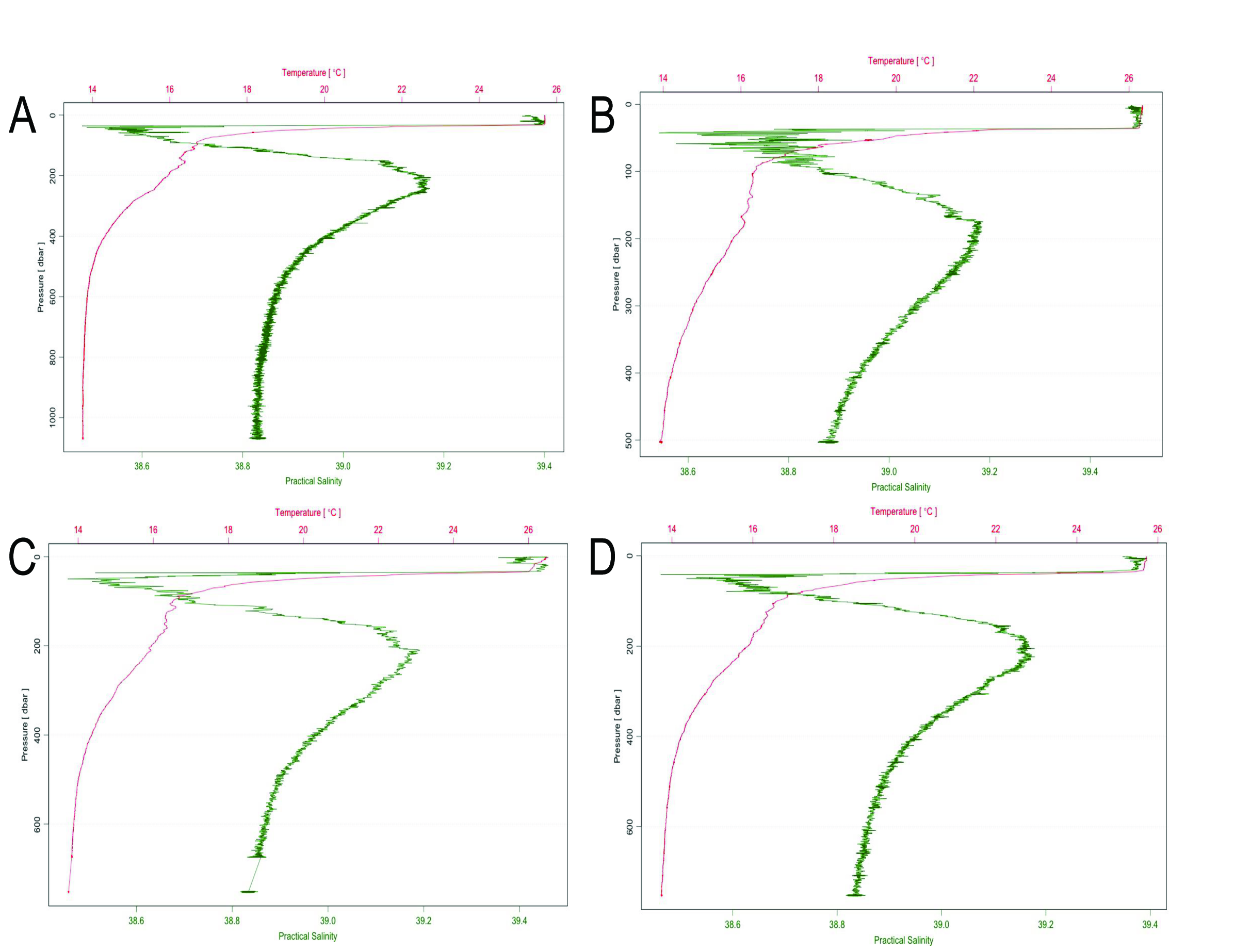

Supplement: S2 Fig — (A) Total Nitrogen (B) Nitrate (C) Inorganic Phosphate (D) Ammonia (E) NPOC (F) Silicate (G) Sulfate (H) Iron. Shapes represent different sampling stations. Colors represent water masses. (TIF) [file pone.0120605.s002.tif]

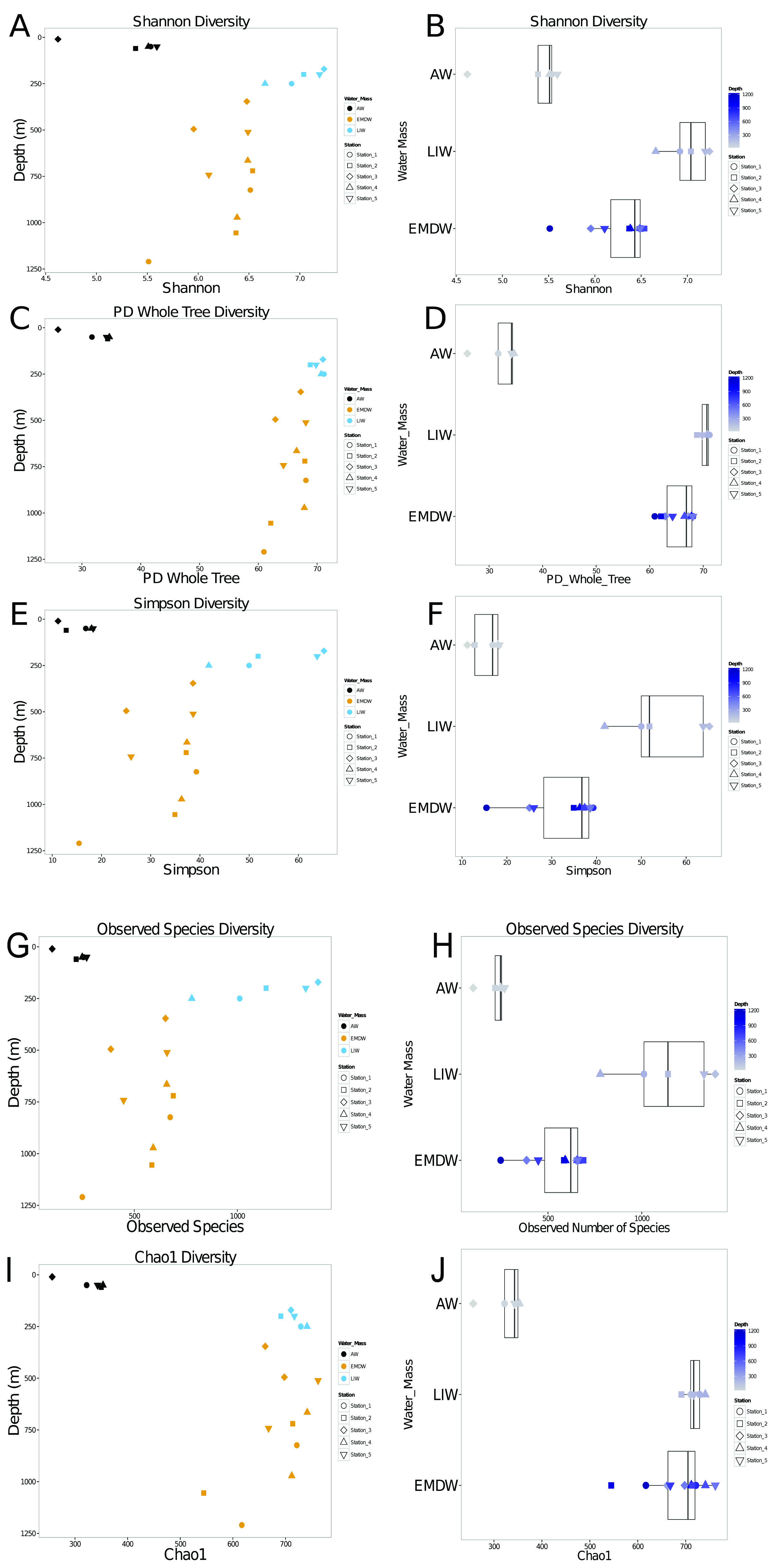

Supplement: S3 Fig — (A) Shannon diversity plotted as a function of depth. Symbols represent sampling location and colors represent water masses. (B) Shannon diversity for each water mass. Symbols represent sampling locations. Colors correspond to depth. (C) PD Whole Tree diversity plotted as a function of depth. Symbols represent sampling location and colors represent water masses. (D) PD Whole Tree diversity for each water mass. Symbols represent sampling locations. Colors correspond to depth. (E) Simpson diversity plotted as a function of depth. Symbols represent sampling location and colors represent water masses. (F) Simpson diversity for each water mass. Symbols represent sampling locations. Colors correspond to depth. (TIF) [file pone.0120605.s003.tif]

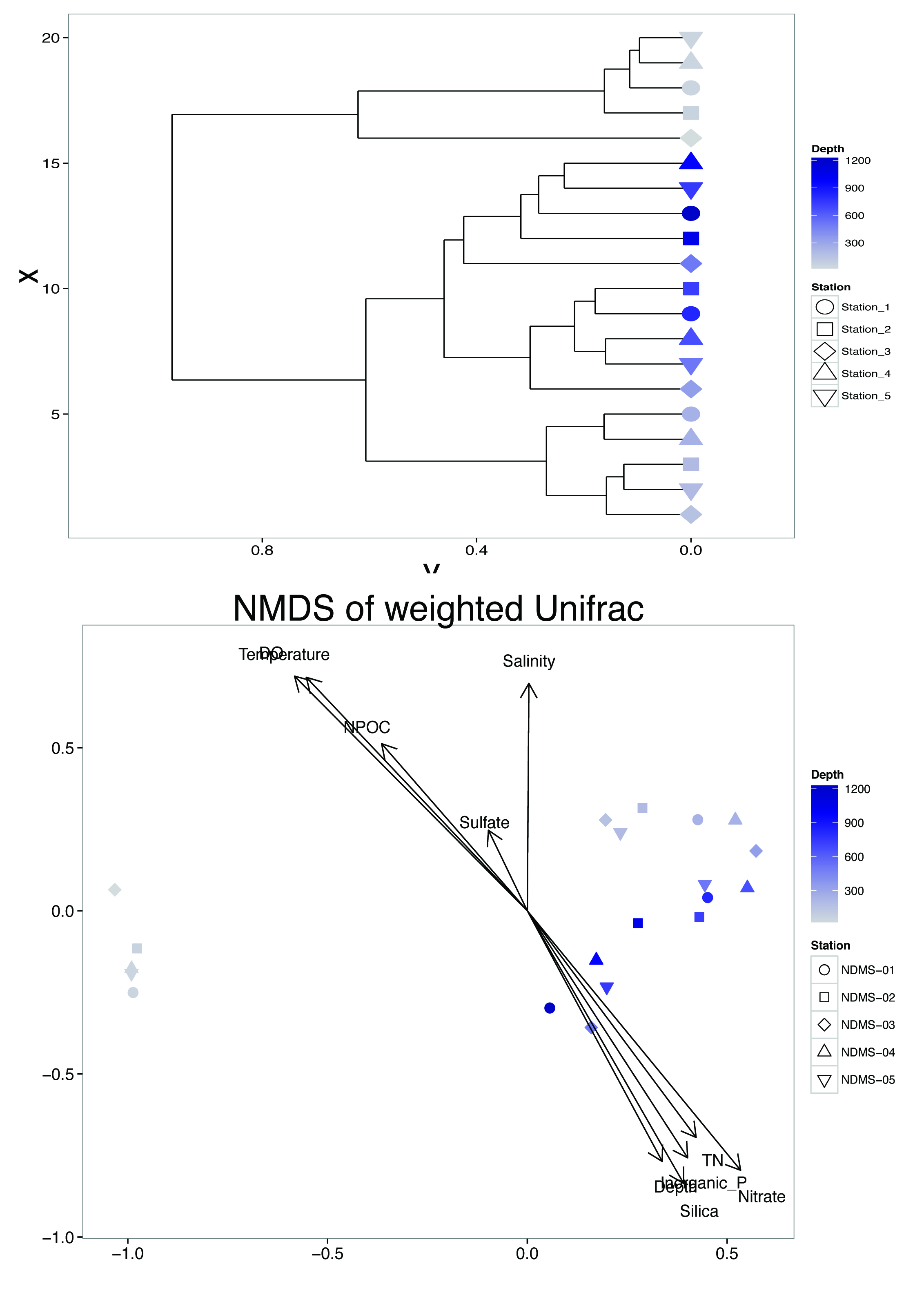

Supplement: S4 Fig — (A) Hierarchichal Clustering Analysis. Symbols represent sampling station and colors correspond to depth. (B)Non-metric multidimensional scaling analysis of weighted unifrac distances. Geochemical variable were fit to the weighted unifrac distance matric. Vectors are shown for environmental variables that fit the data with a p value of greater than 0.01. Shapes represent sampling station. Colors correspond to depth. (TIF) [file pone.0120605.s004.tif]

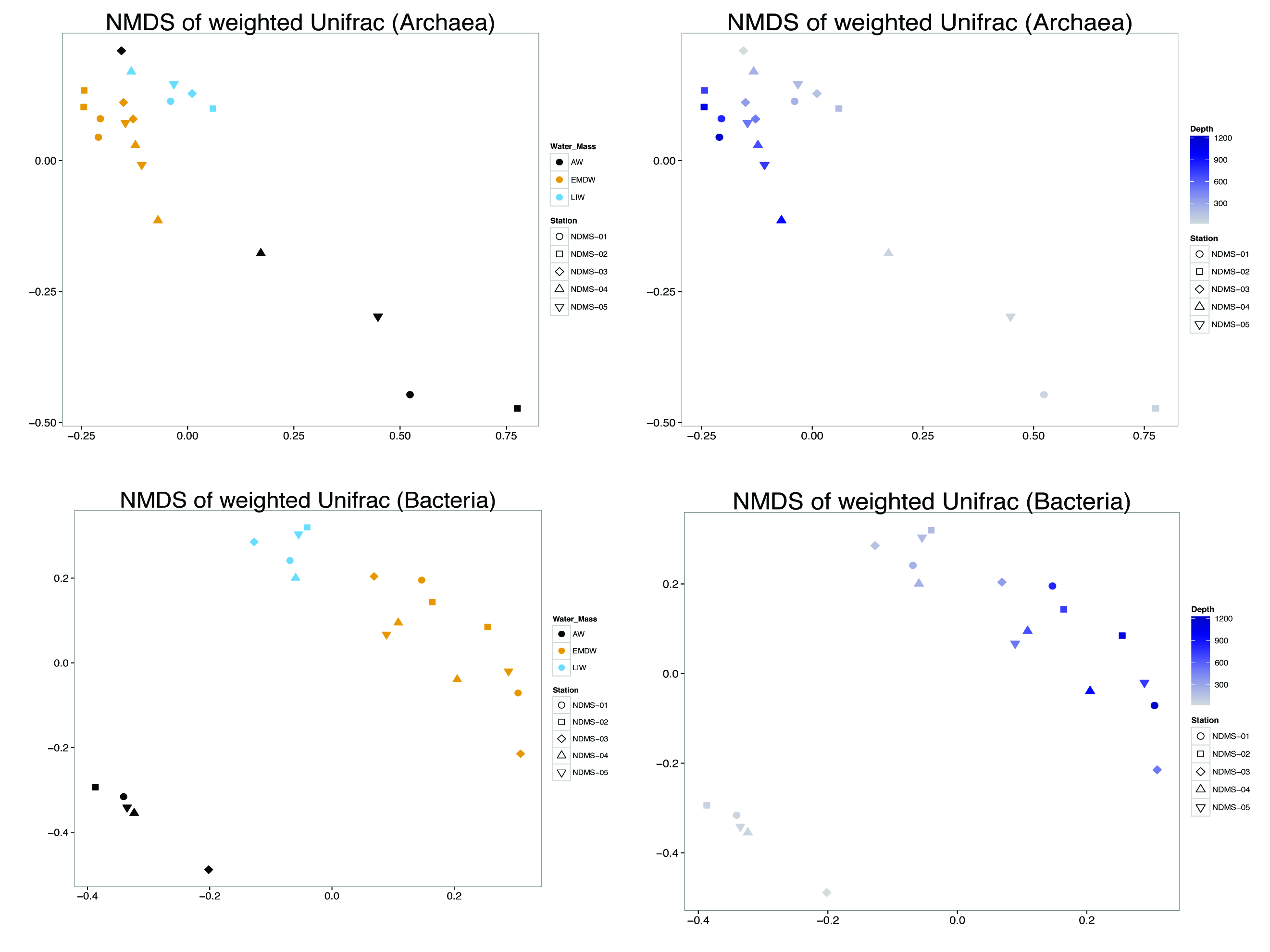

Supplement: S5 Fig — (A and B) NMDS plot of weighted unifrac distances determined for an OTU table of OTUs classified as Archaea. (A) Symbols represent sampling station and colors represent water mass (B) symbols represent sampling station colors correspond to depth. (C and D) NMDS plot of weighted unifrac distances determined for an OTU table of OTUs classified as Bacteria. (C) Symbols represent sampling station and colors represent water mass (D) symbols represent sampling station colors correspond to depth. (TIF) [file pone.0120605.s005.tif]

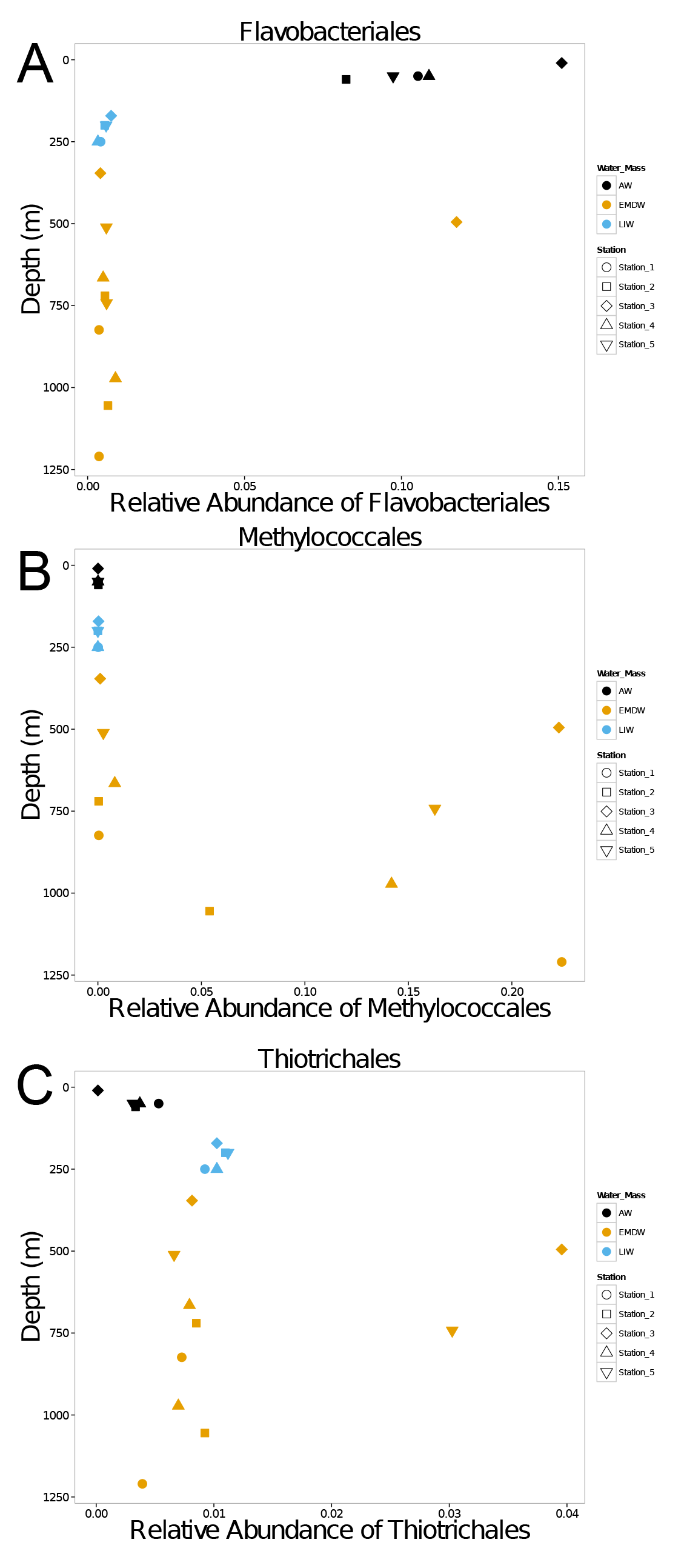

Supplement: S6 Fig — Three microbial classes were highly enriched in the water sample directly above the North Alex Mud Volcano. (A) Relative abundance of Flavobacteriales plotted as a function of depth. Symbols represent sampling location. Colors represent water mass. (B) Relative abundance of Methylococcales plotted as a function of depth. Symbols represent sampling location. Colors represent water mass. (C) Relative abundance of Thiotrichales plotted as a function of depth. Symbols represent sampling location. Colors represent water mass. (TIF) [file pone.0120605.s006.tif]
