# Supplementary material for: The Unique Chemistry of Eastern Mediterranean Water Masses Selects for Distinct Microbial Communities by Depth
Source: PLoS One. 2015 Mar 25;10(3):e0120605. doi: 10.1371/journal.pone.0120605 (PMC4373936; doi:10.1371/journal.pone.0120605)
Supplement: S1 Table — (DOCX) [file pone.0120605.s007.docx]

**S1 Table.**

| **Sample ID** | **Station** | **Depth** | **Volume Filtered** | **Temp** | **Salinity** | **DO** | **pH** | **Turbidity** | **Sulfate** | **Fe** | **Inorganic Phosphate** | **Silicate** | **Ammonia** | **Nitrate** | **Total N** | **TOC** | **Water Mass** |
| --- | --- | --- | --- | --- | --- | --- | --- | --- | --- | --- | --- | --- | --- | --- | --- | --- | --- |
|  |  | **(m)** | **L** | **°C** | **psu** | **% Sat** |  | **FTU** | **mg/L** | **µg/L** | **µmol/L** | **µmol/L** | **µmol/L** | **µmol/L** | **µmol/L** | **NPOC (μM)** |  |
| NDMS001 | 3 | 10 | 70 | 26.33 | 39.49 | 102.86 | 8.2 | 1.31 | 3486 | <5.0 | 0.171 | 0.58 | <0.02 | <0.01 | 7.23 | 100.67 | AW |
| NDMS009 | 1 | 50 | 97.5 | 19.20 | 38.63 | 115.82 | 8.2 | 1.31 | 3406 | 5.4 | 0.104 | 0.43 | <0.02 | <0.01 | 7.08 | 99.17 |  |
| NDMS014 | 4 | 50 | 100.2 | 19.28 | 38.56 | 127.52 | 8.2 | 1.19 | 6544 | <5.0 | 0.063 | 0.41 | <0.02 | <0.01 | 7.32 | 92.92 |  |
| NDMS024 | 5 | 50 | 90.1 | 19.00 | 38.60 | 116.38 | 8.2 | 1.25 | 7042 | <5.0 | <0.008 | 0.38 | <0.02 | <0.01 | 7.72 | 103.58 |  |
| NDMS019 | 2 | 60 | 95.7 | 17.49 | 38.59 | 111.80 | 8.2 | 1.25 | 7183 | <5.0 | 0.033 | 0.51 | <0.02 | <0.01 | 7.23 | 90.84 |  |
| NDMS003 | 3 | 171 | 81.9 | 16.04 | 39.14 | 91.61 | 8.2 | 1.31 | 3134 | <5.0 | 0.160 | 1.39 | <0.02 | 1.924 | 7.08 | 80.20 | LIW |
| NDMS018 | 2 | 200 | 74.1 | 15.88 | 39.18 | 91.13 | 8.2 | 1.25 | 7107 | <5.0 | 0.069 | 1.36 | <0.02 | 2.091 | 8.26 | 82.41 |  |
| NDMS023 | 5 | 200 | 72.9 | 15.81 | 39.16 | 89.93 | 8.2 | 1.19 | 4417 | <5.0 | 0.057 | 1.84 | <0.02 | 2.572 | 8.75 | 76.88 |  |
| NDMS008 | 1 | 250 | 75 | 15.57 | 39.15 | 87.78 | 8.2 | 1.31 | 3119 | 6.8 | 0.240 | 2.74 | <0.02 | 3.383 | 10.21 | 88.51 |  |
| NDMS013 | 4 | 250 | 68.7 | 15.54 | 39.16 | 88.72 | 8.2 | 1.19 | 6509 | <5.0 | 0.168 | 2.61 | <0.02 | 3.396 | 8.11 | 61.56 |  |
| NDMS004 | 3 | 346 | 126.3 | 14.48 | 38.99 | 76.37 | 8.2 | 1.31 | 3270 | <5.0 | 0.375 | 6.65 | <0.02 | 6.315 | 11.90 | 123.40 | EMDW |
| NDMS002 | 3 | 495 | 99.3 | 13.96 | 38.88 | 70.50 | 8.2 | 1.38 | 2978 | 6.1 | 0.437 | 8.81 | <0.02 | 6.776 | 11.56 | 74.49 |  |
| NDMS022 | 5 | 511 | 93.3 | 13.94 | 38.88 | 73.17 | 8.2 | 1.19 | 6831 | <5.0 | 0.299 | 8.78 | <0.02 | 6.953 | 11.03 | 72.66 |  |
| NDMS012 | 4 | 665 | 102.6 | 13.84 | 38.85 | 70.60 | 8.2 | 1.25 | 7243 | 5 | 0.367 | 8.97 | <0.028 | 6.59 | 10.09 | 57.96 |  |
| NDMS017 | 2 | 720 | 92.4 | 13.82 | 38.84 | 70.66 | 8.2 | 1.25 | 4703 | <5.0 | 0.338 | 9.33 | 0.131 | 6.659 | 13.86 | 60.45 |  |
| NDMS021 | 5 | 742 | 62.1 | 13.75 | 38.83 | 69.19 | 8.2 | 1.31 | 5523 | <5.0 | 0.298 | 9.74 | <0.02 | 6.315 | 10.81 | 68.89 |  |
| NDMS007 | 1 | 824 | 158.1 | 13.77 | 38.83 | 69.35 | 8.2 | 1.31 | 3300 | 6.1 | 0.381 | 9.64 | <0.02 | 6.495 | 9.42 | 56.64 |  |
| NDMS011 | 4 | 972 | 72.9 | 13.75 | 38.83 | 67.65 | 8.2 | 1.25 | 4055 | 9.5 | 0.354 | 9.57 | <0.02 | 5.856 | 10.18 | 61.89 |  |
| NDMS016 | 2 | 1055 | 84.1 | 13.76 | 38.83 | 66.59 | 8.2 | 1.25 | 3884 | <5.0 | 0.327 | 9.99 | <0.02 | 6.382 | 9.89 | 62.46 |  |
| NDMS006 | 1 | 1210 | 69.3 | 13.79 | 38.83 | 65.43 | 8.2 | 1.31 | 2174 | <5.0 | 0.371 | 9.81 | 0.108 | 5.987 | 10.21 | 70.92 |  |
