# Supplementary material for: The Unique Chemistry of Eastern Mediterranean Water Masses Selects for Distinct Microbial Communities by Depth
Source: PLoS One. 2015 Mar 25;10(3):e0120605. doi: 10.1371/journal.pone.0120605 (PMC4373936; doi:10.1371/journal.pone.0120605)
Supplement: S2 Table — Mole percent for each lipid detected in each samples. Groups of samples are shown according to the water mass from which they were obtained. In the sample names, the first number corresponds to the sampling station and the second number is the unique identifier for that sample. The average of each lipid in each water mass is shown as well as the minimum and maximum mole percent values for each lipid in each water mass. (DOCX) [file pone.0120605.s008.docx]

**S2 Table.**

| **FAME** | **03-001** | **01-009** | **04-014** | **05-024** | **02-019** | **AW Average** | **AW Min** | **AW Max** |
| --- | --- | --- | --- | --- | --- | --- | --- | --- |
| **Depth** | 10 | 50 | 50 | 50 | 60 |  |  |  |
| 14:1w5 | 0.00 | 0.00 | 0.00 | 0.00 | 0.00 | 0.00 | 0.00 | 0.00 |
| 14:0 | 15.23 | 13.45 | 14.00 | 13.74 | 15.78 | 14.10 | 13.45 | 15.23 |
| i15:0 | 0.64 | 0.49 | 0.57 | 0.47 | 0.33 | 0.54 | 0.47 | 0.64 |
| a15:0 | 0.34 | 0.41 | 0.41 | 0.40 | 0.33 | 0.39 | 0.34 | 0.41 |
| 15:0 | 1.03 | 0.98 | 1.16 | 1.13 | 0.93 | 1.07 | 0.98 | 1.16 |
| 10me15:0 | 0.00 | 0.00 | 0.10 | 0.00 | 0.00 | 0.02 | 0.00 | 0.10 |
| 16:2w6 | 0.00 | 0.00 | 0.00 | 0.00 | 0.00 | 0.00 | 0.00 | 0.00 |
| 16:1w9 | 0.00 | 0.00 | 0.30 | 0.24 | 0.00 | 0.14 | 0.00 | 0.30 |
| 16:1w9/8 | 0.00 | 0.00 | 0.00 | 0.00 | 0.00 | 0.00 | 0.00 | 0.00 |
| 16:1w7 | 0.00 | 12.59 | 13.56 | 13.09 | 13.63 | 9.81 | 0.00 | 13.56 |
| 16:1 | 0.00 | 0.62 | 0.77 | 0.64 | 0.56 | 0.51 | 0.00 | 0.77 |
| 16:0 | 34.35 | 23.85 | 25.41 | 26.19 | 24.04 | 27.45 | 23.85 | 34.35 |
| 10me16:0 | 0.00 | 0.16 | 0.21 | 0.21 | 0.00 | 0.15 | 0.00 | 0.21 |
| i17:0 | 1.47 | 0.00 | 0.00 | 0.00 | 0.00 | 0.37 | 0.00 | 1.47 |
| cyc17:0 | 1.43 | 4.97 | 6.59 | 6.09 | 4.62 | 4.77 | 1.43 | 6.59 |
| 17:0 | 0.00 | 0.00 | 0.85 | 0.88 | 0.00 | 0.43 | 0.00 | 0.88 |
| 18:4w3 | 0.97 | 1.92 | 1.62 | 1.93 | 2.62 | 1.61 | 0.97 | 1.93 |
| 18:2w6 | 2.10 | 1.78 | 1.51 | 1.61 | 1.54 | 1.75 | 1.51 | 2.10 |
| 18:3w3 | 0.97 | 1.21 | 1.08 | 1.32 | 1.57 | 1.15 | 0.97 | 1.32 |
| 18:1w9 | 3.92 | 3.80 | 3.45 | 3.27 | 3.01 | 3.61 | 3.27 | 3.92 |
| 18:1w9/8 | 9.50 | 10.47 | 12.78 | 12.58 | 9.62 | 11.33 | 9.50 | 12.78 |
| 18:1w5 | 0.00 | 0.00 | 1.01 | 0.00 | 0.00 | 0.25 | 0.00 | 1.01 |
| 18:0 | 5.17 | 2.73 | 2.30 | 2.64 | 1.75 | 3.21 | 2.30 | 5.17 |
| 10me18:0 | 0.00 | 0.00 | 0.00 | 0.00 | 0.00 | 0.00 | 0.00 | 0.00 |
| cyc19:0 | 1.13 | 1.39 | 1.74 | 1.63 | 1.07 | 1.47 | 1.13 | 1.74 |
| 20:5w3 | 2.68 | 0.63 | 0.00 | 0.00 | 0.00 | 0.83 | 0.00 | 2.68 |
| 20:0 | 0.77 | 3.64 | 0.36 | 0.43 | 4.04 | 1.30 | 0.36 | 3.64 |
| 22:1 | 0.00 | 0.31 | 0.00 | 0.00 | 0.26 | 0.08 | 0.00 | 0.31 |
| 22:6w3 | 12.49 | 0.00 | 9.65 | 10.75 | 0.00 | 8.22 | 0.00 | 12.49 |
| 23:0 | 0.00 | 14.58 | 0.00 | 0.00 | 14.31 | 3.65 | 0.00 | 14.58 |
| 24:0 | 0.00 | 0.00 | 0.59 | 0.76 | 0.00 | 0.34 | 0.00 | 0.76 |

| **FAME** | **03-003** | **05-023** | **02-018** | **01-008** | **04-013** | **LIW Average** | **LIW Min** | **LIW Max** |
| --- | --- | --- | --- | --- | --- | --- | --- | --- |
| **Depth (m)** | 171 | 200 | 200 | 250 | 250 |  |  |  |
| 14:1w5 | 0.00 | 0.00 | 0.00 | 0.00 | 0.00 | 0.00 | 0.00 | 0.00 |
| 14:0 | 3.44 | 3.31 | 1.68 | 2.96 | 2.54 | 2.81 | 1.68 | 3.44 |
| i15:0 | 0.82 | 1.07 | 0.67 | 0.90 | 0.96 | 0.85 | 0.67 | 1.07 |
| a15:0 | 0.00 | 0.70 | 0.50 | 0.52 | 0.66 | 0.40 | 0.00 | 0.70 |
| 15:0 | 1.00 | 1.04 | 1.08 | 0.75 | 0.94 | 1.04 | 1.00 | 1.08 |
| 10me15:0 | 0.00 | 0.00 | 0.00 | 0.00 | 0.00 | 0.00 | 0.00 | 0.00 |
| 16:2w6 | 0.00 | 0.00 | 0.00 | 0.00 | 0.00 | 0.00 | 0.00 | 0.00 |
| 16:1w9 | 0.00 | 0.00 | 0.00 | 0.64 | 0.00 | 0.00 | 0.00 | 0.00 |
| 16:1w9/8 | 0.00 | 0.00 | 0.00 | 0.00 | 0.00 | 0.00 | 0.00 | 0.00 |
| 16:1w7 | 15.51 | 14.58 | 13.62 | 12.27 | 13.83 | 14.57 | 13.62 | 15.51 |
| 16:1 | 0.87 | 1.09 | 1.00 | 0.95 | 1.24 | 0.99 | 0.87 | 1.09 |
| 16:0 | 24.15 | 22.90 | 27.74 | 24.46 | 24.75 | 24.93 | 22.90 | 27.74 |
| 10me16:0 | 1.52 | 1.73 | 1.74 | 1.45 | 1.80 | 1.66 | 1.52 | 1.74 |
| i17:0 | 0.00 | 0.00 | 0.00 | 0.00 | 0.00 | 0.00 | 0.00 | 0.00 |
| cyc17:0 | 13.02 | 14.16 | 15.15 | 15.02 | 15.95 | 14.11 | 13.02 | 15.15 |
| 17:0 | 0.00 | 0.00 | 0.92 | 1.24 | 0.00 | 0.31 | 0.00 | 0.92 |
| 18:4w3 | 0.00 | 0.00 | 0.00 | 0.00 | 0.00 | 0.00 | 0.00 | 0.00 |
| 18:2w6 | 0.00 | 0.00 | 0.00 | 0.00 | 0.00 | 0.00 | 0.00 | 0.00 |
| 18:3w3 | 0.00 | 0.00 | 0.00 | 0.00 | 0.00 | 0.00 | 0.00 | 0.00 |
| 18:1w9 | 5.11 | 3.86 | 3.79 | 3.17 | 4.69 | 4.25 | 3.79 | 5.11 |
| 18:1w9/8 | 14.32 | 12.55 | 1.33 | 11.25 | 13.11 | 9.40 | 1.33 | 14.32 |
| 18:1w5 | 0.00 | 0.00 | 0.00 | 0.00 | 0.00 | 0.00 | 0.00 | 0.00 |
| 18:0 | 7.45 | 7.73 | 7.64 | 4.56 | 5.76 | 7.61 | 7.45 | 7.73 |
| 10me18:0 | 3.83 | 0.00 | 5.57 | 3.21 | 0.00 | 3.13 | 0.00 | 5.57 |
| cyc19:0 | 5.07 | 4.73 | 5.16 | 5.88 | 4.58 | 4.99 | 4.73 | 5.16 |
| 20:5w3 | 0.00 | 0.00 | 2.67 | 1.74 | 0.00 | 0.89 | 0.00 | 2.67 |
| 20:0 | 0.00 | 2.73 | 1.27 | 0.96 | 2.28 | 1.33 | 0.00 | 2.73 |
| 22:1 | 0.00 | 0.00 | 0.00 | 0.00 | 0.00 | 0.00 | 0.00 | 0.00 |
| 22:6w3 | 0.00 | 0.00 | 5.49 | 3.70 | 0.00 | 1.83 | 0.00 | 5.49 |
| 23:0 | 3.89 | 7.81 | 0.00 | 0.00 | 6.92 | 3.90 | 0.00 | 7.81 |
| 24:0 | 0.00 | 0.00 | 2.97 | 4.36 | 0.00 | 0.99 | 0.00 | 2.97 |

| **FAME** | **03-004** | **03-002** | **05-022** | **04-012** | **02-017** | **05-021** | **01-007** | **04-011** | **02-016** | **01-006** | **EMDW Average** | **EMDW Min** | **EMDW Max** |
| --- | --- | --- | --- | --- | --- | --- | --- | --- | --- | --- | --- | --- | --- |
| **Depth (m)** | 346 | 495 | 511 | 665 | 720 | 742 | 824 | 972 | 1055 | 1210 |  |  |  |
| 14:1w5 | 0.00 | 0.00 | 0.00 | 0.00 | 0.00 | 0.00 | 0.00 | 0.00 | 0.00 | 1.19 | 0.00 | 0.00 | 0.00 |
| 14:0 | 5.80 | 1.37 | 0.64 | 4.20 | 0.95 | 0.88 | 4.90 | 0.00 | 2.50 | 0.70 | 2.31 | 0.64 | 5.80 |
| i15:0 | 1.47 | 0.51 | 0.29 | 0.00 | 0.92 | 0.29 | 1.49 | 0.00 | 0.82 | 0.25 | 0.58 | 0.00 | 1.47 |
| a15:0 | 1.21 | 0.34 | 0.51 | 0.00 | 0.89 | 0.27 | 1.49 | 0.00 | 0.55 | 0.22 | 0.54 | 0.00 | 1.21 |
| 15:0 | 1.80 | 0.63 | 0.00 | 0.00 | 0.88 | 0.29 | 0.00 | 0.00 | 0.77 | 0.22 | 0.60 | 0.00 | 1.80 |
| 10me15:0 | 0.23 | 0.66 | 0.00 | 0.00 | 0.00 | 0.00 | 0.00 | 0.00 | 2.67 | 0.00 | 0.15 | 0.00 | 0.66 |
| 16:2w6 | 0.00 | 0.00 | 0.00 | 0.00 | 0.00 | 2.87 | 0.00 | 0.00 | 0.00 | 4.68 | 0.48 | 0.00 | 2.87 |
| 16:1w9 | 1.18 | 0.00 | 0.00 | 0.00 | 0.00 | 0.00 | 0.00 | 0.00 | 0.00 | 0.00 | 0.20 | 0.00 | 1.18 |
| 16:1w9/8 | 0.00 | 50.56 | 3.07 | 6.91 | 0.00 | 48.40 | 0.00 | 53.03 | 26.36 | 42.32 | 18.16 | 0.00 | 50.56 |
| 16:1w7 | 11.14 | 16.67 | 8.85 | 12.70 | 11.72 | 24.94 | 12.68 | 21.83 | 13.90 | 21.39 | 14.34 | 8.85 | 24.94 |
| 16:1 | 1.01 | 0.44 | 0.00 | 0.00 | 0.00 | 0.46 | 0.00 | 0.00 | 0.57 | 0.26 | 0.32 | 0.00 | 1.01 |
| 16:0 | 32.93 | 13.21 | 34.97 | 33.91 | 24.09 | 9.31 | 28.47 | 9.27 | 21.74 | 10.75 | 24.74 | 9.31 | 34.97 |
| 10me16:0 | 1.62 | 0.46 | 1.46 | 0.00 | 2.40 | 0.64 | 0.00 | 0.70 | 1.06 | 0.34 | 1.10 | 0.00 | 2.40 |
| i17:0 | 0.00 | 0.00 | 0.00 | 0.00 | 0.00 | 0.00 | 0.00 | 0.00 | 0.00 | 0.00 | 0.00 | 0.00 | 0.00 |
| cyc17:0 | 11.95 | 3.08 | 12.97 | 13.60 | 19.39 | 4.28 | 16.35 | 4.32 | 4.84 | 2.37 | 10.88 | 3.08 | 19.39 |
| 17:0 | 1.45 | 0.59 | 0.00 | 0.00 | 0.00 | 0.00 | 0.00 | 0.00 | 1.27 | 0.65 | 0.34 | 0.00 | 1.45 |
| 18:4w3 | 0.00 | 0.00 | 0.00 | 0.00 | 0.00 | 0.00 | 0.00 | 0.00 | 0.00 | 0.00 | 0.00 | 0.00 | 0.00 |
| 18:2w6 | 0.42 | 0.20 | 0.00 | 0.00 | 0.00 | 0.00 | 0.00 | 0.00 | 0.00 | 0.31 | 0.10 | 0.00 | 0.42 |
| 18:3w3 | 0.00 | 0.00 | 0.00 | 0.00 | 0.00 | 0.00 | 0.00 | 0.00 | 0.00 | 0.00 | 0.00 | 0.00 | 0.00 |
| 18:1w9 | 2.70 | 1.25 | 3.80 | 0.00 | 5.26 | 0.69 | 4.12 | 1.54 | 2.09 | 1.23 | 2.28 | 0.00 | 5.26 |
| 18:1w9/8 | 7.79 | 3.62 | 8.56 | 7.64 | 14.75 | 3.61 | 9.25 | 4.60 | 4.45 | 3.39 | 7.66 | 3.61 | 14.75 |
| 18:1w5 | 0.00 | 0.00 | 0.00 | 0.00 | 0.00 | 0.00 | 0.00 | 0.00 | 0.00 | 1.11 | 0.00 | 0.00 | 0.00 |
| 18:0 | 7.52 | 1.90 | 12.16 | 8.32 | 7.75 | 2.18 | 14.19 | 3.82 | 7.58 | 2.54 | 6.64 | 1.90 | 12.16 |
| 10me18:0 | 1.41 | 0.35 | 1.04 | 0.00 | 3.12 | 0.00 | 3.03 | 0.00 | 0.00 | 0.54 | 0.99 | 0.00 | 3.12 |
| cyc19:0 | 2.80 | 1.24 | 3.40 | 4.65 | 5.90 | 0.89 | 4.03 | 0.90 | 1.42 | 0.72 | 3.15 | 0.89 | 5.90 |
| 20:5w3 | 0.00 | 0.72 | 1.55 | 0.00 | 0.00 | 0.00 | 0.00 | 0.00 | 0.00 | 0.00 | 0.38 | 0.00 | 1.55 |
| 20:0 | 1.09 | 0.33 | 1.27 | 0.00 | 0.00 | 0.00 | 0.00 | 0.00 | 1.37 | 0.58 | 0.45 | 0.00 | 1.27 |
| 22:1 | 0.00 | 0.00 | 0.00 | 0.00 | 1.97 | 0.00 | 0.00 | 0.00 | 0.00 | 0.00 | 0.33 | 0.00 | 1.97 |
| 22:6w3 | 1.87 | 0.67 | 3.49 | 1.85 | 0.00 | 0.00 | 0.00 | 0.00 | 0.00 | 0.00 | 1.31 | 0.00 | 3.49 |
| 23:0 | 0.00 | 0.00 | 0.00 | 0.00 | 0.00 | 0.00 | 0.00 | 0.00 | 0.00 | 0.00 | 0.00 | 0.00 | 0.00 |
| 24:0 | 2.58 | 1.22 | 1.97 | 6.20 | 0.00 | 0.00 | 0.00 | 0.00 | 6.03 | 4.26 | 2.00 | 0.00 | 6.20 |
