# Supplementary material for: The Unique Chemistry of Eastern Mediterranean Water Masses Selects for Distinct Microbial Communities by Depth
Source: PLoS One. 2015 Mar 25;10(3):e0120605. doi: 10.1371/journal.pone.0120605 (PMC4373936; doi:10.1371/journal.pone.0120605)
Supplement: S3 Table — The sample station, depth and water mass for each sample are also shown for each sample. (DOCX) [file pone.0120605.s009.docx]

**S3 Table.**

| **Sample** | **MG-RAST ID** | **Number of Sequences** | **Station** | **Water Mass** | **Depth (m)** |
| --- | --- | --- | --- | --- | --- |
| NDMS001 | 4571952.3 | 92493 | Station 3 | AW | 10 |
| NDMS009 | 4571959.3 | 53320 | Station 1 | AW | 50 |
| NDMS014 | 4571963.3 | 84623 | Station 4 | AW | 50 |
| NDMS024 | 4571971.3 | 160639 | Station 5 | AW | 50 |
| NDMS019 | 4571967.3 | 122322 | Station 2 | AW | 60 |
| NDMS003 | 4571954.3 | 61808 | Station 3 | LIW | 171 |
| NDMS018 | 4571966.3 | 121597 | Station 2 | LIW | 200 |
| NDMS023 | 4571970.3 | 118040 | Station 5 | LIW | 200 |
| NDMS008 | 4571958.3 | 71652 | Station 1 | LIW | 250 |
| NDMS013 | 4571962.3 | 101120 | Station 4 | LIW | 250 |
| NDMS004 | 4571955.3 | 32423 | Station 3 | EMDW | 346 |
| NDMS002 | 4571953.3 | 71437 | Station 3 | EMDW | 495 |
| NDMS022 | 4571969.3 | 148360 | Station 5 | EMDW | 511 |
| NDMS012 | 4571961.3 | 76611 | Station 4 | EMDW | 665 |
| NDMS017 | 4571965.3 | 83592 | Station 2 | EMDW | 720 |
| NDMS021 | 4571968.3 | 80427 | Station 5 | EMDW | 742 |
| NDMS007 | 4571957.3 | 43833 | Station 1 | EMDW | 824 |
| NDMS011 | 4571960.3 | 91330 | Station 4 | EMDW | 972 |
| NDMS016 | 4571964.3 | 13753 | Station 2 | EMDW | 1055 |
| NDMS006 | 4571956.3 | 46323 | Station 1 | EMDW | 1210 |
