# Supplementary material for: The Unique Chemistry of Eastern Mediterranean Water Masses Selects for Distinct Microbial Communities by Depth
Source: PLoS One. 2015 Mar 25;10(3):e0120605. doi: 10.1371/journal.pone.0120605 (PMC4373936; doi:10.1371/journal.pone.0120605)
Supplement: S4 Table — Two-way ANOVA and Tukey tests were used to compare the relative abundance of microbial classes in order to determine which classes were significantly different between the three water masses. ANOVA F statistic and p value are shown for each taxa. p values were corrected using the false discovery rate calculation in R. Tukey test was used to determine which water masses were significantly different from each other for that class. The mean relative abundance of each class is also reported for each water mass. (DOCX) [file pone.0120605.s010.docx]

|  | **ANOVA** | | **Tukey** | | | **Relative Abundance** | | |
| --- | --- | --- | --- | --- | --- | --- | --- | --- |
| **Taxa** | **F Statistic** | **corrected p value** | **EMDW-AW** | **LIW-AW** | **LIW-EMDW** | **AW Mean** | **LIW Mean** | **EMDW Mean** |
| Archaea [Parvarchaeota] [Parvarchaea] | 18.62 | 9.59E-05 | 8.18E-05 | 0.375767456 | 0.002222419 | 0 | 0.0002 | 0.000879808 |
| Archaea Crenarchaeota MBGA | 84.51 | 2.01E-08 | 0.00047 | 1.33E-09 | 6.04E-08 | 0 | 0.00492 | 0.001614193 |
| Archaea Crenarchaeota Thaumarchaeota | 53.26 | 1.43E-07 | 9.20E-08 | 3.02E-07 | 0.825726146 | 0.000407184 | 0.35 | 0.333389079 |
| Archaea Euryarchaeota Halobacteria | 28.09 | 9.32E-06 | 0.998 | 3.00E-05 | 5.63E-06 | 0 | 0.0016 | 1.45E-05 |
| Archaea Euryarchaeota Thermoplasmata | 68.10 | 5.45E-08 | 2.77E-08 | 2.53E-08 | 0.244860058 | 0.001439686 | 0.13 | 0.111364793 |
| ArchaeaOtherOther | 7.35 | 0.00634 | 0.0159 | 1 | 0.015907345 | 0 | 0 | 0.00063986 |
| Bacteria [Thermi] Deinococci | 10.81 | 0.00137 | 1 | 0.003908962 | 0.001120046 | 0 | 0.00266 | 0 |
| Bacteria Acidobacteria Acidobacteria-6 | 15.89 | 0.00022 | 0.00535 | 8.74E-05 | 0.029235425 | 2.91E-05 | 0.0300 | 0.016912674 |
| Bacteria Acidobacteria AT-s2-57 | 14.70 | 0.00032 | 0.00498 | 0.000141837 | 0.054104835 | 0 | 0.0009 | 0.00050898 |
| Bacteria Acidobacteria BPC102 | 53.26 | 1.43E-07 | 9.20E-08 | 3.02E-07 | 0.825726146 | 0 | 0.00188 | 0.002719407 |
| Bacteria Acidobacteria PAUC37f | 9.36 | 0.00253 | 0.1758 | 0.001436827 | 0.018850546 | 0 | 0.00031 | 0.000116338 |
| Bacteria Acidobacteria Solibacteres | 4.53 | 0.03082 | 0.0291 | 0.696552534 | 0.171379577 | 0 | 4.36E-05 | 0.000130881 |
| Bacteria Acidobacteria Sva0725 | 9.09 | 0.00281 | 0.0076 | 1 | 0.007638077 | 0 | 0 | 0.000196321 |
| Bacteria Actinobacteria Acidimicrobiia | 3.87 | 0.04620 | 0.7805 | 0.047435797 | 0.080354781 | 0.048 | 0.022075184 | 0.042252599 |
| Bacteria AncK6 | 53.26 | 1.43E-07 | 9.20E-08 | 3.02E-07 | 0.825726146 | 0 | 0.00096 | 7.27E-06 |
| Bacteria Bacteroidetes [Rhodothermi] | 12.07 | 0.00082 | 0.00074 | 0.002047525 | 0.986567848 | 0.0042 | 1.45E-05 | 0.000152694 |
| Bacteria Bacteroidetes [Saprospirae] | 53.26 | 1.43E-07 | 9.20E-08 | 3.02E-07 | 0.825726146 | 0.00050898 | 0 | 4.36E-05 |
| Bacteria Bacteroidetes Cytophagia | 13.79 | 0.00042 | 0.00079 | 0.882385358 | 0.002558263 | 0.0021 | 0.0019 | 0.000327201 |
| Bacteria Bacteroidetes Flavobacteriia | 21.34 | 4.55E-05 | 5.18E-05 | 7.08E-05 | 0.750904597 | 0.109 | 0.0052 | 0.016672726 |
| Bacteria Bacteroidetes Sphingobacteriia | 18.16 | 0.00011 | 8.93E-05 | 0.000278942 | 0.973110775 | 0.0021 | 2.91E-05 | 0.000109067 |
| Bacteria BacteroidetesOther | 4.01 | 0.04281 | 0.0455 | 0.809912181 | 0.1704444 | 0 | 0.000159965 | 0.000581691 |
| Bacteria Chloroflexi Anaerolineae | 26.19 | 1.36E-05 | 0.665 | 1.99E-05 | 1.60E-05 | 1.45E-05 | 0.0042 | 0.00050898 |
| Bacteria Chloroflexi Ktedonobacteria | 13.99 | 0.00041 | 0.95819 | 0.000924945 | 0.000388182 | 0 | 0.0020 | 0.000109067 |
| Bacteria Chloroflexi SAR202 | 80.61 | 2.01E-08 | 7.56E-05 | 1.47E-09 | 2.78E-07 | 0.000174507 | 0.07482 | 0.029462663 |
| Bacteria Chloroflexi TK17 | 53.26 | 1.43E-07 | 9.20E-08 | 3.02E-07 | 0.825726146 | 0 | 0.0031 | 0.000305388 |
| Bacteria ChloroflexiOther | 41.92 | 7.63E-07 | 0.00081 | 1.71E-07 | 4.18E-05 | 0 | 0.0044 | 0.00191231 |
| Bacteria Cyanobacteria Chloroplast | 427.83 | 1.32E-13 | 1.66E-13 | 2.55E-13 | 0.57406097 | 0.008958046 | 0.00073 | 0.000414455 |
| Bacteria Cyanobacteria Synechococcophycideae | 404.21 | 1.32E-13 | 1.71E-13 | 2.45E-13 | 0.908321445 | 0.363717007 | 0.00409 | 0.009648804 |
| Bacteria Gemmatimonadetes Gemm-2 | 28.69 | 8.46E-06 | 0.00011 | 2.83E-06 | 0.017710028 | 1.45E-05 | 0.0073 | 0.004675344 |
| Bacteria Gemmatimonadetes Gemm-4 | 53.26 | 1.43E-07 | 9.20E-08 | 3.02E-07 | 0.825726146 | 0 | 0.0006 | 0.000428997 |
| Bacteria Lentisphaerae [Lentisphaeria] | 6.36 | 0.01073 | 0.9128 | 0.0159894 | 0.013644219 | 0 | 0.000901621 | 0.000101796 |
| Bacteria Nitrospirae Nitrospira | 56.52 | 1.43E-07 | 1.08E-07 | 0.36 | 1.48E-06 | 0 | 0.0011 | 0.006449502 |
| Bacteria PAUC34f | 32.78 | 3.90E-06 | 0.089 | 1.91E-06 | 1.35E-05 | 4.36E-05 | 0.0019 | 0.000516251 |
| Bacteria Planctomycetes BD7-11 | 53.26 | 1.43E-07 | 9.20E-08 | 3.02E-07 | 0.825726146 | 0 | 1.45E-05 | 0.000479895 |
| Bacteria Planctomycetes OM190 | 27.41 | 1.05E-05 | 1.66E-13 | 2.55E-13 | 0.57406097 | 0.00028 | 0.0020 | 0.008565404 |
| Bacteria Planctomycetes Phycisphaerae | 19.16 | 8.37E-05 | 3.99E-05 | 0.089549261 | 0.009020929 | 0.002 | 0.0076 | 0.014782229 |
| Bacteria Planctomycetes Pla3 | 29.90 | 6.72E-06 | 1.77E-06 | 0.00027 | 0.180990959 | 0 | 0.0023 | 0.003010252 |
| Bacteria Planctomycetes Planctomycetia | 25.14 | 1.70E-05 | 0.156 | 1.08E-05 | 6.30E-05 | 0.0017 | 0.0384 | 0.01096488 |
| Bacteria Proteobacteria Alphaproteobacteria | 234.79 | 7.89E-12 | 6.55E-13 | 1.28E-11 | 0.411898783 | 0.196626191 | 0.04547 | 0.0353741 |
| Bacteria Proteobacteria Deltaproteobacteria | 81.87 | 2.01E-08 | 6.16E-06 | 1.10E-09 | 1.56E-06 | 0.00663128 | 0.07306 | 0.038108049 |
| Bacteria Proteobacteria Gammaproteobacteria | 53.26 | 1.43E-07 | 9.20E-08 | 3.02E-07 | 0.825726146 | 0.209554279 | 0.091 | 0.229099106 |
| Bacteria ProteobacteriaOther | 53.26 | 1.43E-07 | 9.20E-08 | 3.02E-07 | 0.825726146 | 0.001614193 | 0.00081 | 0.001694176 |
| Bacteria SAR406 AB16 | 32.65 | 3.90E-06 | 1.66E-13 | 2.55E-13 | 0.57406097 | 0.012259143 | 0.0393 | 0.054279066 |
| Bacteria SBR1093 A712011 | 6.19 | 0.01158 | 0.0435 | 0.008586943 | 0.404595469 | 0 | 0.000567149 | 0.000378099 |
| Bacteria Verrucomicrobia [Pedosphaerae] | 8.23 | 0.00409 | 0.0027 | 0.267088775 | 0.117799874 | 0 | 0.000276303 | 0.000588962 |
| Bacteria Verrucomicrobia Opitutae | 9.89 | 0.00202 | 0.0013 | 0.008956511 | 0.927980871 | 0.019 | 0.00270 | 0.001192467 |
| Bacteria Verrucomicrobia Verruco-5 | 5.26 | 0.01980 | 0.0153 | 0.474504311 | 0.207388034 | 0.00095 | 0.000596234 | 0.000145423 |
| Bacteria Verrucomicrobia Verrucomicrobiae | 53.26 | 1.43E-07 | 9.20E-08 | 3.02E-07 | 0.825726146 | 0 | 0.0050 | 0.000428997 |
| Bacteria ZB3 BS119 | 16.77 | 0.00016 | 6.43E-05 | 0.004249564 | 0.348090805 | 0.0019 | 0.0005 | 4.36E-05 |
| Bacteria Other Other | 69.50 | 5.35E-08 | 0.00017 | 4.54E-09 | 7.97E-07 | 0.001410601 | 0.02607 | 0.011117574 |
| Unclassified Other Other | 8.28 | 0.00408 | 0.0106 | 1 | 0.010644584 | 0 | 0 | 0.000661674 |
