# Supplementary material for: The Unique Chemistry of Eastern Mediterranean Water Masses Selects for Distinct Microbial Communities by Depth
Source: PLoS One. 2015 Mar 25;10(3):e0120605. doi: 10.1371/journal.pone.0120605 (PMC4373936; doi:10.1371/journal.pone.0120605)
Supplement: S5 Table — (DOCX) [file pone.0120605.s011.docx]

| **Taxon** | **Water Mass** | **IndVal** | **Corrected p-value** |
| --- | --- | --- | --- |
| k Archaea p Euryarchaeota c Halobacteria o Halobacteriales f Halobacteriaceae Other | LIW | 0.99 | 0.019 |
| k Archaea p Euryarchaeota c [Parvarchaea] o WCHD3 30 f g | EMDW | 0.81 | 0.019 |
| k Archaea p Crenarchaeota c MBGA o f g | LIW | 0.79 | 0.019 |
| k Archaea p Crenarchaeota c Thaumarchaeota o Cenarchaeales f Cenarchaeaceae Other | LIW | 0.78 | 0.019 |
|  |  |  |  |
| **Taxon** | **Water Mass** | **IndVal** | **Corrected p-value** |
| k Bacteria p Proteobacteria c Gammaproteobacteria o Oceanospirillales f SUP05 g | EMDW | 0.99 | 0.019 |
| k Bacteria p SAR406 c AB16 o Arctic96B 7 f A714017 g SHAG537 | EMDW | 0.99 | 0.019 |
| k Bacteria p Proteobacteria c Alphaproteobacteria o Rhodobacterales f Rhodobacteraceae g | AW | 0.99 | 0.019 |
| k Bacteria p Proteobacteria c Gammaproteobacteria o Methylococcales f Methylococcaceae g | EMDW | 0.99 | 0.019 |
| k Bacteria p Proteobacteria c Gammaproteobacteria o Alteromonadales f Alteromonadaceae g HTCC2207 | AW | 0.98 | 0.019 |
| k Bacteria p Verrucomicrobia c Opitutae o Puniceicoccales f Puniceicoccaceae g Coraliomargarita | AW | 0.98 | 0.019 |
| k Bacteria p Verrucomicrobia c Verruco 5 o R76 B128 f g | AW | 0.97 | 0.019 |
| k Bacteria p Cyanobacteria Other Other Other Other | AW | 0.97 | 0.019 |
| k Bacteria p Cyanobacteria c Synechococcophycideae o Synechococcales f Synechococcaceae g Prochlorococcus | AW | 0.97 | 0.019 |
| k Bacteria p Proteobacteria c Deltaproteobacteria o Sva0853 f S25 1238 g | AW | 0.97 | 0.019 |
| k Bacteria p Bacteroidetes c Sphingobacteriia o Sphingobacteriales f Balneolaceae g | AW | 0.97 | 0.020 |
| k Bacteria p Verrucomicrobia c Verrucomicrobiae o Verrucomicrobiales f Verrucomicrobiaceae g | LIW | 0.96 | 0.019 |
| k Bacteria p Proteobacteria c Betaproteobacteria o MWH UniP1 f g | AW | 0.96 | 0.019 |
| k Bacteria p Proteobacteria c Alphaproteobacteria o Rhodobacterales f Rhodobacteraceae Other | AW | 0.96 | 0.019 |
| k Bacteria p Proteobacteria c Gammaproteobacteria o Alteromonadales f OM60 g | AW | 0.96 | 0.019 |
| k Bacteria p Chloroflexi c TK17 o f g | LIW | 0.96 | 0.019 |
| k Bacteria p Bacteroidetes c Flavobacteriia o Flavobacteriales f Flavobacteriaceae g | AW | 0.95 | 0.019 |
| k Bacteria p Proteobacteria c Alphaproteobacteria o Rickettsiales f AEGEAN 112 g | AW | 0.94 | 0.019 |
| k Bacteria p Verrucomicrobia c Verrucomicrobiae o Verrucomicrobiales f Verrucomicrobiaceae g MSBL3 | LIW | 0.94 | 0.020 |
| k Bacteria p Actinobacteria c Acidimicrobiia o Acidimicrobiales f OCS155 g | AW | 0.92 | 0.023 |
| k Bacteria p Actinobacteria c Acidimicrobiia o Acidimicrobiales f TK06 g | LIW | 0.87 | 0.019 |
| k Bacteria p Planctomycetes c Planctomycetia o Planctomycetales f Planctomycetaceae g Planctomyces | LIW | 0.86 | 0.019 |
| k Bacteria p SAR406 c AB16 o Arctic96B 7 f A714017 g | EMDW | 0.86 | 0.019 |
| k Bacteria p Proteobacteria c Deltaproteobacteria o NB1 j Other Other | EMDW | 0.86 | 0.019 |
| k Bacteria p Proteobacteria c Gammaproteobacteria o Oceanospirillales f Halomonadaceae g Candidatus Portiera | AW | 0.85 | 0.019 |
| k Bacteria p Chloroflexi c TK17 o TK18 f g | LIW | 0.85 | 0.019 |
| k Bacteria p Bacteroidetes c Flavobacteriia o Flavobacteriales f Flavobacteriaceae Other | AW | 0.84 | 0.019 |
| k Bacteria p Proteobacteria c Deltaproteobacteria o Desulfobacterales f Nitrospinaceae g Nitrospina | LIW | 0.82 | 0.019 |
| k Bacteria p Nitrospirae c Nitrospira o Nitrospirales f Nitrospiraceae g | EMDW | 0.81 | 0.019 |
| k Bacteria p Proteobacteria c Alphaproteobacteria o Rickettsiales f g | AW | 0.81 | 0.019 |
| k Bacteria p Planctomycetes c OM190 o agg27 f g | EMDW | 0.80 | 0.019 |
| k Bacteria p Proteobacteria c Deltaproteobacteria o Sva0853 Other Other | LIW | 0.78 | 0.020 |
| k Bacteria p Chloroflexi Other Other Other Other | LIW | 0.76 | 0.019 |
